# *TCF21* and the environmental sensor aryl-hydrocarbon receptor cooperate to activate a pro-inflammatory gene expression program in coronary artery smooth muscle cells

**DOI:** 10.1371/journal.pgen.1006750

**Published:** 2017-05-08

**Authors:** Juyong Brian Kim, Milos Pjanic, Trieu Nguyen, Clint L. Miller, Dharini Iyer, Boxiang Liu, Ting Wang, Olga Sazonova, Ivan Carcamo-Orive, Ljubica Perisic Matic, Lars Maegdefessel, Ulf Hedin, Thomas Quertermous

**Affiliations:** 1 Division of Cardiovascular Medicine, Stanford University, Stanford, California, United States of America; 2 Cardiovascular Institute, Stanford University, Stanford, California, United States of America; 3 Department of Biology, Stanford University, Stanford, California, United States of America; 4 Department of Molecular Medicine and Surgery, Karolinska Institute, Solna, Sweden; Geisel School of Medicine at Dartmouth, UNITED STATES

## Abstract

Both environmental factors and genetic loci have been associated with coronary artery disease (CAD), however gene-gene and gene-environment interactions that might identify molecular mechanisms of risk are not easily studied by human genetic approaches. We have previously identified the transcription factor *TCF21* as the causal CAD gene at 6q23.2 and characterized its downstream transcriptional network that is enriched for CAD GWAS genes. Here we investigate the hypothesis that TCF21 interacts with a downstream target gene, the aryl hydrocarbon receptor (AHR), a ligand-activated transcription factor that mediates the cellular response to environmental contaminants, including dioxin and polycyclic aromatic hydrocarbons (e.g., tobacco smoke). Perturbation of *TCF21* expression in human coronary artery smooth muscle cells (HCASMC) revealed that TCF21 promotes expression of *AHR*, its heterodimerization partner *ARNT*, and cooperates with these factors to upregulate a number of inflammatory downstream disease related genes including *IL1A*, *MMP1*, and *CYP1A1*. TCF21 was shown to bind in *AHR*, *ARNT* and downstream target gene loci, and co-localization was noted for AHR-ARNT and TCF21 binding sites genome-wide in regions of HCASMC open chromatin. These regions of co-localization were found to be enriched for GWAS signals associated with cardio-metabolic as well as chronic inflammatory disease phenotypes. Finally, we show that similar to TCF21, AHR gene expression is increased in atherosclerotic lesions in mice in vivo using laser capture microdissection, and AHR protein is localized in human carotid atherosclerotic lesions where it is associated with protein kinases with a critical role in innate immune response. These data suggest that TCF21 can cooperate with AHR to activate an inflammatory gene expression program that is exacerbated by environmental stimuli, and may contribute to the overall risk for CAD.

## Author summary

Coronary heart disease is the leading cause of death in the world. Both genes and the environment are important risk factors for the progression of disease, however, how genes may modulate the harmful response to the disease promoting environment is unknown and difficult to study. Here, we show that a common heritable variation in the gene TCF21 may regulate coronary heart disease risk by regulating the response of downstream gene activation by the disease environment. We find that a well-known environmental sensor, aryl-hydrocarbon receptor (AHR), is regulated by TCF21 and also interacts with TCF21, resulting in regulation of pro-inflammatory gene expression in coronary artery smooth muscle cells. We further show that oxidized LDL, a well-known driver of atherosclerosis in the plaque can activate the AHR pathway. This work describes a heritable form of gene-environment interaction identified through genome wide association studies in coronary artery disease, and presents an opportunity to define causal gene-gene and gene-environment interactions.

## Introduction

Genome-wide association studies (GWAS) have identified susceptibility loci and candidate genetic variants that predispose to atherosclerotic coronary artery disease (CAD) in humans.[1–4] Despite significant advances made in mapping the genetic contribution to CAD, there has been limited progress toward understanding molecular mechanisms leading to increased atherosclerosis susceptibility that are mediated through gene-environment (GxE) interactions.[5] The difficulty in identifying the role of genetic variation in the differential response to environmental exposure stems from inaccurate quantification of the exposure, the inability to isolate the exposures of interest, and the lack of statistical power.[6]

GWA studies have identified variation at 6q23.2 to be associated with CAD in Caucasian and Han Chinese populations[1, 7], and work in this lab has identified *TCF21* as the causal gene in this locus.[8, 9] Mechanistic studies employing lineage tracing in murine disease models have found that *Tcf21* expression is localized to the medial and adventitial layers of the coronary vessel wall at baseline, and that *Tcf21* expressing cells migrate through the lesion and contribute to the fibrous cap as disease progresses.[10] These data, in combination with in vitro studies indicating that *TCF21* inhibits differentiation and promotes SMC proliferation, suggest a role for this transcription factor in the phenotypic modulation of medial SMC in the response to vascular injury.[10, 11] Further, our RNA-seq and ChIP-seq studies have shown that TCF21 binds and regulates a network of genes associated with CAD.[12] We discovered one of the central components of the *TCF21* gene network to be the aryl hydrocarbon receptor (AHR), a transcription factor that mediates the response to environmental toxins and xenobiotics, and is known to regulate the inflammatory cellular response.[13–17]

AHR binds to a complex array of nuclear proteins involved in diverse processes related to signaling through hormone receptor and inflammatory pathways, chromatin remodeling, etc., and activates a number of target genes, including cytochromes P450 (*CYP1A1* and *CYP1B1*), and AHR repressor (*AHRR*).[18, 19] *AHR* is active primarily in the liver, however it is also strongly expressed in the cardiovascular system, where it has been described to play a role in the cardiovascular development and vascular remodeling.[20–22] In the context of environmental stimuli, AHR ligands include a wide range of environmental pollutants, including 2,3,7,8-tetrachlorodibenzo-p-dioxin (dioxin), co-planar polychlorinated biphenyls (PCBs), and polycyclic aromatic hydrocarbons (PAH) which are major constituents of tobacco smoke.[15, 22–24]

The correlation of major cardiovascular risk factors with the *AHR* pathway relates to epidemiological evidence that dioxin exposure is linked to increased cardiovascular mortality.[25] Furthermore, murine model studies have shown that mice carrying an AHR variant with higher ligand affinity developed more severe atherosclerosis compared to wild-type mice[22], and an increase in disease burden when exposed to dioxin.[26] In humans, a common SNP associated with *AHR* was found to correlate with the CAD phenotype in a Chinese population.[27] In addition, the expression level of *AHR* in circulating peripheral mononuclear cells was associated with acute coronary syndromes (ACS), suggesting that greater *AHR* level might be associated with plaque instability and rupture.

Given the role of *AHR* in mediating inflammation and atherosclerosis, we postulated that *TCF21* may alter the risk of atherosclerosis by modulating the AHR pathway. We set out to characterize the intersection of these pathways at the genomic level, identify the possible mechanisms of interaction, and to determine the role of TCF21-AHR interactions in the context of inflammation in the vessel wall. Through these studies, we define the molecular mechanisms by which these two transcriptional pathways interact to regulate the risk of atherosclerosis.

## Results

### *AHR* is a TCF21 targeted gene differentially regulated in SMC

We have previously reported the analysis of an in vitro si*TCF21* knockdown RNA-seq study in human coronary artery smooth muscle cells (HCASMC) and noted differential expression of a number of inflammatory genes and pathways.[10] Interestingly, this module included the gene encoding *AHR* which directs an inflammatory program as part of its repertoire of response to xenobiotics[13, 14, 26], and the xenobiotic pathway was identified as one of those differentially regulated with *TCF21* modulation.[10] Also, ChIP-seq studies in HCASMC have shown TCF21 binding in the AHR locus, suggesting that this gene is regulated in part by TCF21, and raising our interest in possible interactions between these transcriptional networks.[12]

While *AHR* has not been associated with CAD risk using the statistical criteria employed for GWAS efforts, we have identified a variant within the *AHR* locus (rs608646) that has a nominal association (p = 0.0047) in the CARDIOGRAM+C4D GWAS data in the context of a single SNP study (S1A Fig). This SNP was also noted to regulate expression of *AHR* as identified with eQTL studies in GTEx tissues high in SMC content (S1B Fig). In aortic and coronary artery tissues, the *AHR* locus (+/- 1Mb) was generally enriched with *AHR-*eQTL signals with multiple peaks uniformly scattered throughout the locus (S2 Fig), indicating that *AHR* gene expression is genetically regulated in the vasculature and emphasizing the importance of *AHR* in these tissues. These data thus validate a previous candidate gene study that found association of CAD with *AHR* in East Asians.[26]

### TCF21 regulates inflammatory target genes, including *AHR*, as well as genes that modulate SMC cell state

**Fig 1:**
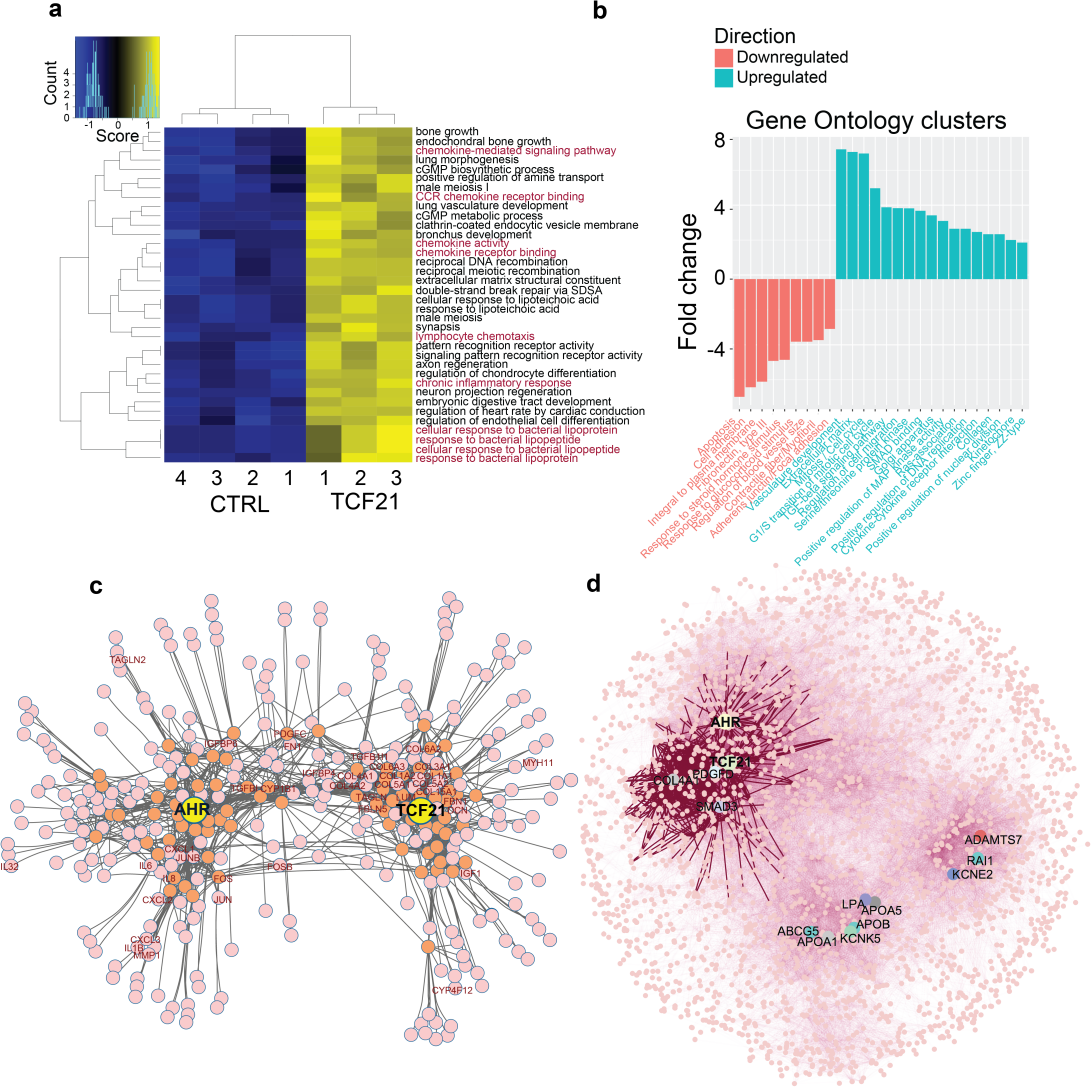
*TCF21* overexpression perturbs expression of chronic inflammatory genes and genes linked to vascular disease pathophysiology. **(**a). Representation of GO categories that are significantly altered by the *TCF21* overexpression. Red text indicates immune related GO terms, black text indicates other categories. CTRL, control cell growth conditions; *TCF21*, *TCF21* over-expression. (b) Selected DAVID GO ontology analysis restricted to genes that show significant up- or down- regulation with *TCF21* overexpression in HCASMC. Upregulated GO clusters colored in blue, downregulated in red. (c) Co-expression modules of *TCF21* and *AHR-ARNT* show a high degree of connectivity. *TCF21* and *AHR* co-expression modules were defined using 4164 human microarray datasets through GeneFriends and visualized with Cytoscape. A few representative genes are identified by gene symbol. (d) Co-expression network of 77 CAD GWAS related genes was built using GeneFriends:Microarray, and visualized with Cytoscape. Transcription modules identify three primary clusters of CAD GWAS genes, with *TCF21* and *AHR* appearing in the same cluster. Genes *PDGFD*, *SMAD3* and *COL4A1* also appear in this cluster. A second cluster is composed primarily of lipid-related genes, such as APOA1, APOA5, APOB, LPA, etc.

To further investigate overlap of these pathways, we sought to expand the repertoire of TCF21 regulated genes by performing a transcriptome analysis with lentivirus mediated *TCF21* over-expression in HCASMC. Analysis of the top 500 differentially regulated genes with *goseq*[28] (Fig 1A, S1 Table) identified a significant number of terms related to embryonic development (*lung morphogenesis*, *lung vasculature development*), but also numerous terms related to innate immunity and inflammation (*response to bacterial lipopeptide*, *response to lipoteichoic acid*, *CCR chemokine receptor binding*, *chemokine receptor binding*, *lymphocyte chemotaxis*, *chronic inflammatory response*), most of which were upregulated with TCF21 overexpression. Employing the DAVID algorithm we found that downregulated genes were primarily associated with SMC development and phenotype (*regulation of blood vessel size*, *contractile fiber/myofibril)* while upregulated genes were primarily associated with cellular proliferation (*mitosis/cell cycle*, *positive regulation of DNA replication*) (Fig 1B, S2 Table). In addition, GO terms enrichment and PCA analysis performed using goseq yielded multiple immune system and atherosclerosis- related GO terms, implicating TCF21 in HCASMC to promote immune and pro-atherosclerotic-responses (S3 Fig).

### TCF21 and AHR transcriptional networks overlap

To look for relationships between AHR and TCF21 transcriptional networks, we investigated correlations among genes that reside in the co-expression modules of both *TCF21* and *AHR*, using publicly available microarray data sets. We created genome-wide gene expression modules using 4164 human microarray data sets, and used the GeneFriends algorithm that reports the top 5% of co-expressed genes as high order associations, as well as second order indirect, associations.[29] In this analysis, *TCF21* and *AHR* shared a number of indirect associations that link the two modules (Fig 1C). Gene ontology analysis of associations with p<0.05 between *TCF21* and *AHR* networks revealed strong enrichment for inflammation, extracellular matrix modification, and developmental terms (S3 Table). *TCF21* and *AHR* appeared to be highly related when co-expression network was visualized with all other CAD GWAS implicated genes, localizing in a cluster of extracellular matrix gene *COL4A1* and growth factor receptor *PDGFR*, and distinct from a cluster of lipid genes (*LPA*, *APOA5*, *APOB*, *APOA1*) (Fig 1D). Further, we identified the co-expression module for *ARNT*, the heterodimer partner of AHR, and found that it also contains genes indirectly connected to *AHR* and *TCF21* modules (S4 Fig), suggesting functional connectivity between *AHR-ARNT* and *TCF21* co-expressed genes (S3 Table).

### TCF21 regulates *AHR* and *ARNT* expression, and these factors together regulate downstream gene expression

**Fig 2:**
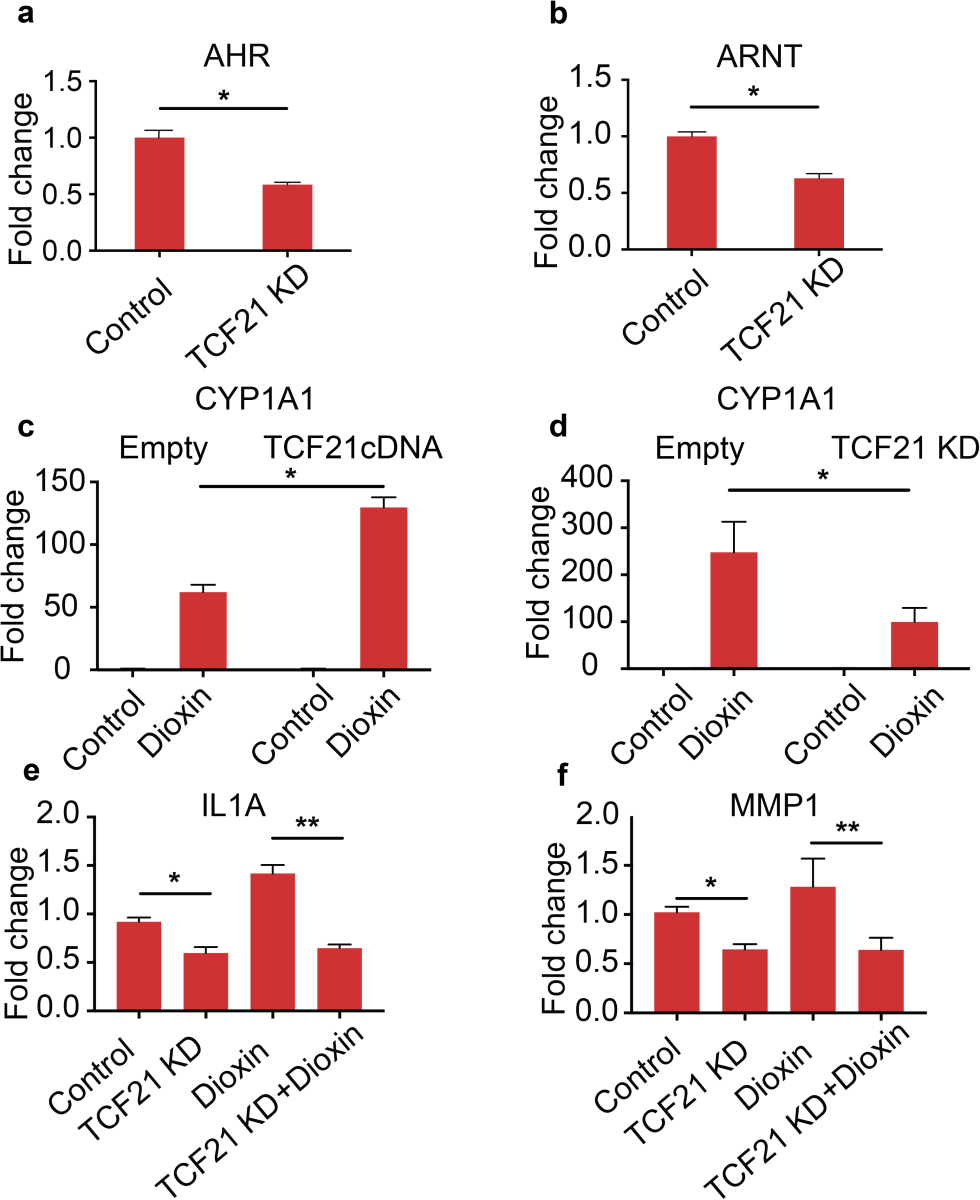
TCF21 modulates expression of *AHR* and its downstream genes. (a, b) Knock-down of *TCF21* reduces expression of *AHR* and *ARNT* in HCASMC. (c, d) Over-expression of *TCF21* further increased expression of *CYP1A1* in HCASMC induced by dioxin treatment, while siRNA mediated knock-down of *TCF21* attenuated expression of *CYP1A1* in HCASMC that was induced by the treatment with dioxin. (e, f) TCF21 knockdown by siRNA decreased expression of both *IL1A* and *MMP1*, while dioxin alone increased expression of both genes. Also, TCF21 knockdown completely blocked the dioxin-mediated increase in expression for both genes.

Experiments were first conducted to determine whether TCF21 modulates expression of *AHR* and *ARNT*. RNA-seq and qPCR analysis in HCASMC showed that the *AHR* and *ARNT* mRNA levels were down-regulated by *TCF21* siRNA knockdown (*AHR* 1.0±0.07 vs. 0.58±0.02, p = 0.0037; and *ARNT* 1.0±0.04 vs. 0.63±0.04, p = 0.0031) (Fig 2A and 2B), and up-regulated by *TCF21* overexpression (S5 Fig). To further characterize the intersection of TCF21 and AHR transcriptional networks, we investigated the mechanism by which TCF21 regulates downstream genes in the AHR pathway, and chose to first study the dioxin effect on the canonical AHR target gene *CYP1A1*. Dioxin induction of *CYP1A1* mRNA levels nearly doubled in HCASMC exposed to both dioxin and *TCF21* transfection compared to dioxin alone (153.5±9.7 vs. 61.8±5.9 fold, P = 0.001), and the opposite result was seen with *TCF21* knock-down in conjunction with dioxin (99.5±29.8 vs. 247.9±64.8, P<0.05) (Fig 2C and 2D). Manipulation of *TCF21* expression alone did not alter *CYP1A1* expression, suggesting that it does not directly affect transcription of this canonical AHR downstream gene, but does alter the response to dioxin most likely through regulation of *AHR* and *ARNT* expression levels.

To investigate the possible interaction of TCF21 and AHR in the regulation of target inflammatory pathway genes, additional studies were conducted in HCASMC. We focused on IL1A and MMP1 genes, as previous studies have found these genes to be representative targets of AHR activation through direct or indirect pathways.[30–32] mRNA levels were measured for *IL1A*, and *MMP1* genes by RT-PCR in HCASMC with *TCF21* expression perturbed by knockdown and over-expression. Knockdown of *TCF21* decreased *IL1A* expression compared to cells treated with scrambled siRNA (0.91±0.04 vs. 0.52±0.06, p = 0.013) (Fig 2E). Dioxin treatment significantly increased expression of *IL1A* (0.91±0.04 vs. 1.42±0.09, p = 0.007), and co-treatment of si*TCF21* blocked this effect (1.42±0.09 vs 0.65±0.04, p = 0.001). Similar results were obtained for *MMP1*, with si*TCF21* alone decreasing gene expression (1.02±0.06 vs. 0.65±0.05, p = 0.009), dioxin increasing expression (1.02±0.06 vs. 1.62±0.28, p = 0.10), and si*TCF21* knocking down the increased expression of *MMP1* seen with dioxin (1.62±0.28 vs. 0.64±0.12, p = 0.034) (Fig 2F).

### TCF21 binds the *AHR* locus and regulates *AHR* expression at the transcriptional level

**Fig 3:**
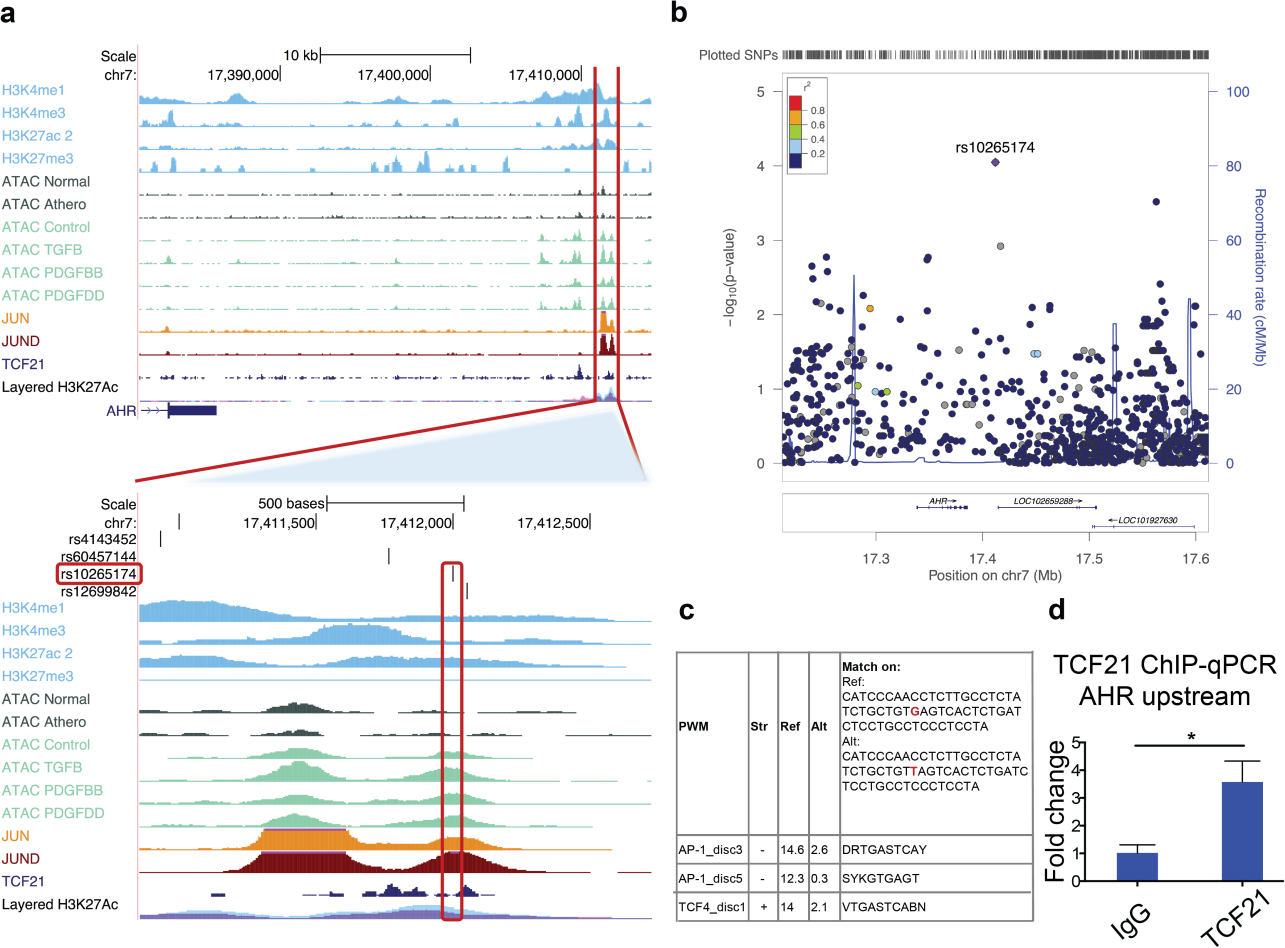
AHR expression in HCASMC is mediated by an eQTL that modulates *TCF21* binding. (a) The downstream region of the *AHR* gene on chr7 (located on >20kb distance from the transcription end site) contains a strong open chromatin region and enhancer in HCASMC marked by ATAC-Seq, H3K27ac tracks and binding of AP1 transcription factors, JUN and JUND, as well as TCF21. Enhanced view shows that SNP rs10265174 directly overlaps the ATAC-Seq open chromatin, H3K27ac enhancer mark, JUN and JUND ChIP-Seq peaks, and is within a broad TCF21 ChIP-Seq peak. (b) The LocusZoom plot shows *AHR* gene eQTL distribution in HCASMC across 1Mb (16.9–17.9Mb) on chr7 encompassing *AHR* gene, with SNP rs10265174 being the top eQTL in the locus. (c) rs10265174 alters the position weight matrix scores for AP1 and TCF4 transcription factors (data from Haploreg). (d) TCF21 ChIP-qPCR shows enrichment at the *AHR* upstream genomic region compared to IgG control (p<0.05).

To begin to investigate the mechanism by which TCF21 regulates *AHR* expression, we correlated whole genome RNA-seq and genotype information developed in 52 HCASMC lines to evaluate expression quantitative trait locus (eQTL) effects at the *AHR* locus.[33] We identified SNP rs10265174 to be one of the top eQTLs for *AHR* (p<9e-5) (Fig 3A and 3B). Also, rs10265174 was consistently found to regulate gene expression in multiple GTEx tissues, including coronary artery, aorta and tibial artery (S4 Table). Furthermore, the SNP was located in an open chromatin region/enhancer marked by ATAC-seq, H3K27ac ChIP-Seq, JUN and JUND ChIP-Seq peaks, and within a TCF21 ChIP-Seq peak. We found the rs10265174 variant to alter the PMW scores for AP1 and TCF4 transcription factors (HaploReg) (Fig 3C). Given that TCF4 is a known bHLH binding partner for TCF21,[34] we evaluated whether TCF21 might directly regulate *AHR* gene expression at this site. We surveyed ChIP-seq data previously generated for TCF21 in HCASMC[12] and identified ChIP-seq peaks representing TCF21 binding sites in both the *AHR* and *ARNT* genes. We confirmed the binding of TCF21 to both genomic regions in HCASMC with ChIP-qPCR for *AHR* (1.02±0.29 vs. 3.58±0.75; p = 0.033) and *ARNT* (1.03±0.28 vs. 20.04±4.80; p = 0.017) loci (Fig 3D, S6 Fig). Taken together, these data suggest that TCF21 may directly regulate expression of both the *AHR* and *ARNT* genes at the transcriptional level.

### TCF21 and AHR-ARNT predicted binding site distributions show genome-wide overlap of target loci

**Fig 4:**
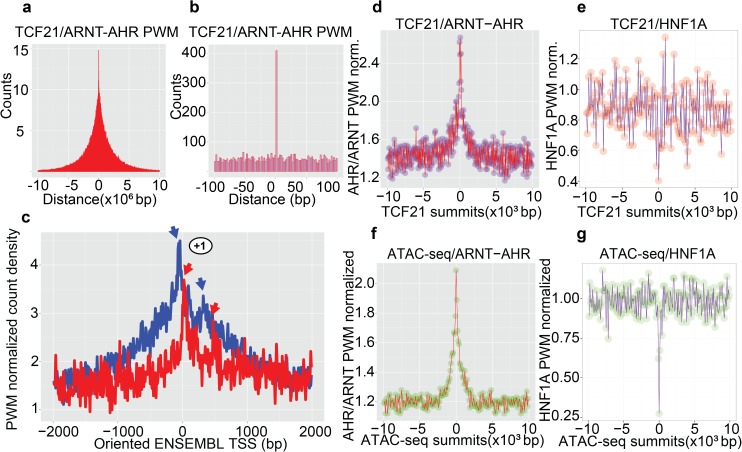
Genome-wide correlation of TCF21 and AHR-ARNT position weight matrices (PWMs). (a) Distribution of distances from the center of AHR-ARNT predicted sites to the closest TCF12/TCF21 predicted site in +/-50kb genomic intervals. TCF12/TCF21 (MA0521.1) and AHR-ARNT (MA0006.1) matrices were obtained from the JASPAR database. (b) Distribution of distances from AHR-ARNT predicted sites to the closest TCF12/TCF21 predicted site in +/-100bp window from the center of the AHR-ARNT sites. The graph shows over 400 overlapping TCF12/TCF21 and AHR-ARNT sites. (c) Genome wide spatial relationship between AHR/ARNT and TCF12/TCF21 PWMs are graphed, and co-localize near transcription start sites (TSSs). Co-localization of AHR/ARNT and TCF12/TCF21 predicted binding sites over oriented ENSEMBL promoters reveals longitudinal phasing of the two matrices with peaks immediately after the transcription start site and also after the +1 nucleosome (arrows). Predicted sites were counted in a window of 10bp and normalized globally to the average number of sites in 10bp windows. (d) Co-localization of AHR/ARNT predicted binding sites and TCF21 ChIP-seq summits is shown as a density plot. Predicted sites were counted in a window of 100bp and normalized globally to the average number of AHR/ARNT sites in 100bp windows. (e) Absence of genome wide correlation between negative control matrix for HNF1A (MA0046.1) and TCF21 summits in HCASMC. Localization of HNF1A predicted binding sites and TCF21 ChIP-seq summits is shown as a density plot. Predicted sites were counted in a window of 100bp and normalized globally to the average number of HNF1A sites in 100bp windows. (f) The genome wide correlation between AHR/ARNT binding sites and open chromatin is shown for HCASMC. Co-localization of AHR/ARNT predicted binding sites and open chromatin ATAC-seq summits is shown as a density plot. (g) The lack of correlation between control HNF1A PWM and open chromatin is shown for HCASMC. Localization of HNF1A predicted binding sites and open chromatin ATAC-seq summits is shown as a density plot.

To further investigate the overlap of TCF21 and AHR transcriptional networks at the genomic level, we determined the genome-wide relationship between binding sites for AHR-ARNT and TCF21. We scanned the human genome sequence with the position weight matrix (PWM) for AHR-ARNT, and scanned for the PWM for TCF12 as a surrogate for TCF21 (JASPAR matrices, Ahr::Arnt—MA0006.1; Tcf12—MA0521.1).[12, 35, 36] TCF12 is the primary heterodimerization partner for TCF21, binds the same primary sequence as TCF21, and this composite site is indicated here as TCF12/TCF21.[12, 36] Predicted binding sites for TCF12/TCF21 and AHR-ARNT identified co-localization within a broad region of 5kb, with 339 high stringency TCF12/TCF21 and AHR-ARNT sites that directly overlap (P<2.2e-16, Fisher exact test, using combined ENCODE open chromatin regions as background; 218 sites overlapping within the background) (Fig 4A and 4B) and 11769 lower stringency sites overlapping (P<2.2e-16, Fisher exact test, ENCODE background; 4833 sites overlapping within the background; S5 Table). Next, we tested the positional orientation of TCF12/TCF21 and AHR-ARNT sites near functional elements, such as promoters, using the collection of 100,276 human ENSEMBL transcription start sites (TSS), Hum_ENSEMBL69 from Biomart. We observed that both matrices show double peaks near the oriented ENSEMBL TSS (Fig 4C). We also noted that these double peaks are in phase with each other, suggesting conservation of spatial orientation between the two sets of predicted binding sites and possible functional interaction of the two proteins that is preserved by evolutionary constraint near the TSS. In addition, we observe that the distance between phased peaks corresponds to the position of the +1 nucleosome, with an additional peak corresponding to the +2 nucleosome in the TCF PWM profile, suggesting that functional interaction between TCF21 and AHR-ARNT would be localized at the boundaries defined by nucleosome positioning at functional regions.

To test whether TCF21 in vivo binding sites correlate with AHR-ARNT PWM predictions, we generated the precise locations of TCF21 ChIP-seq summit positions in HCASMC using the MACS ChIP-seq tool (S6 Table).[37] We found that the center of these TCF21 ChIP-seq summits co-localized with the predicted AHR-ARNT PWM sites, further suggesting that AHR-ARNT complexes co-localize genome-wide with TCF21 in vivo binding sites (Fig 4D). In contrast, control PWMs for kidney/liver specific factors HNF1A and HNF1B showed uniform background distribution near TCF21 summits (Fig 4E and S7 Fig). In addition, AHR-ARNT PWM profiles showed an increase at summits for open chromatin regions in HCASMC, defined with MACS and ATAC-seq HCASMC data sets (Fig 4F). Control HNF1A and HNF1B matrices showed a decrease in their frequency near ATAC-seq summits (Fig 4G and S7 Fig).

**Fig 5:**
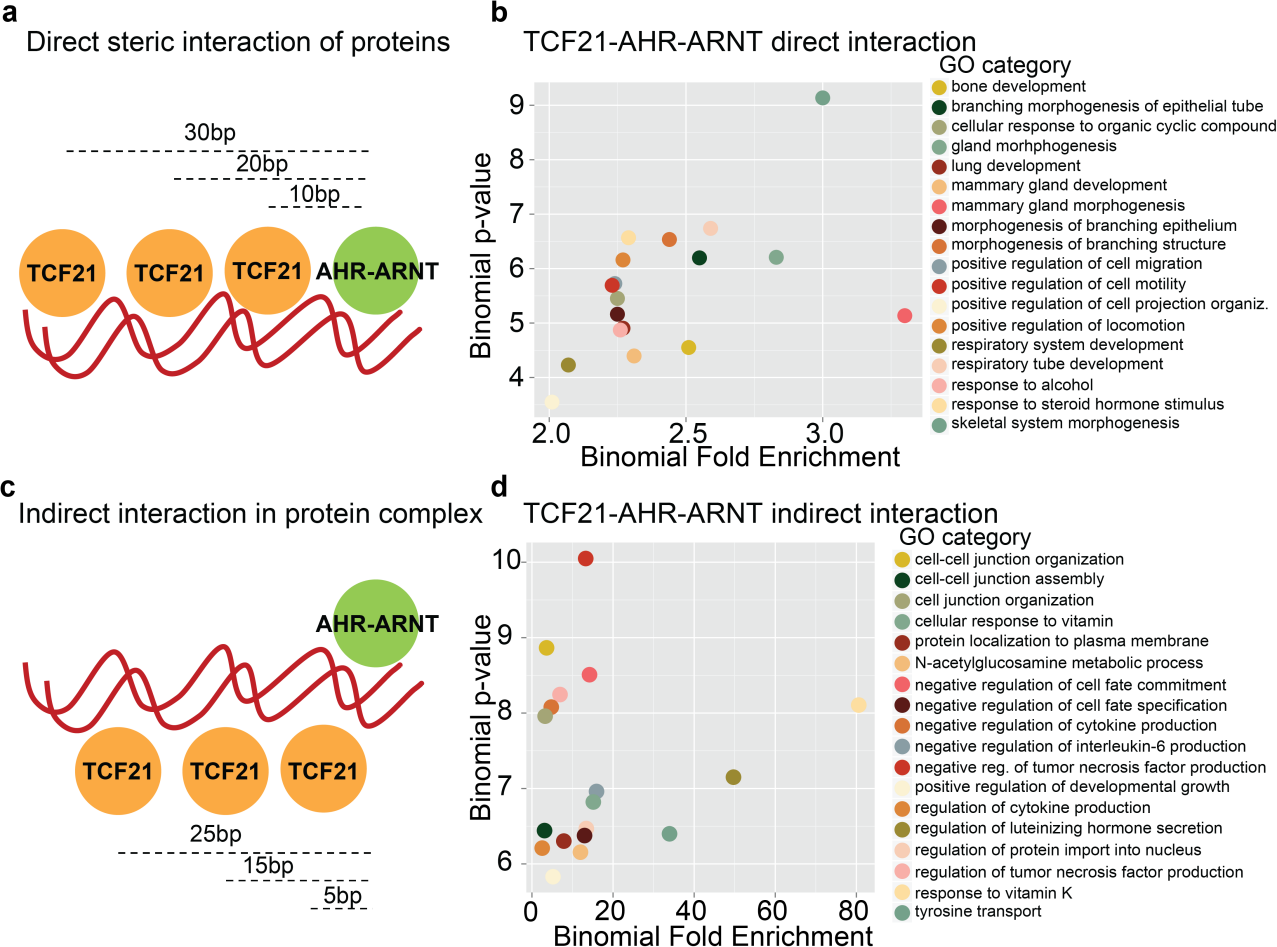
Rotational phasing of PWM predicts steric interaction of TCF21 and AHR differentially regulates transcriptional programs. (a) Schematic representation of rotationally phased TCF21 and AHR spaced at n(10bp) distances along the DNA indicating direct interaction between proteins. (b) GO terms enriched in genes proximal to rotationally phased TCF12, employed here as surrogate for TCF21, and AHR-ARNT JASPAR matrices. (c) Schematic of rotationally unphased TCF21 and AHR proteins spaced at 5bp+n(10bp) distances indicating indirect interaction through an intermediary protein or a protein complex. (d) GO terms enriched in genes proximal to rotationally unphased TCF12 and AHR-ARNT JASPAR matrices.

We further assessed the steric relationship of TCF21 and AHR binding by dividing the co-localized TCF and AHR predicted sites into two categories, rotationally phased and un-phased, as rotational phasing has been shown to be crucial for direct protein binding.[38, 39] We considered PWM sites to be phased if they occurred at distances of n(10bp), i.e. 10, 20, 30, and 40bp, in which case due to the DNA helical pitch they will be oriented on the same side of the DNA strand and capable of direct protein-protein interaction. If separated by distances of 5, 15, 25, 35, 45 bp they would be expected to be oriented on the opposite sides of the DNA molecule due to the pitch of the DNA major groove, rotationally un-phased and less capable of direct protein-protein interaction. In the case of un-phased sites, indirect interaction through a protein complex might still be possible, e.g. through intermediate protein interactions. We extracted genes that are proximal to both categories of sites and calculated GO enrichment using GREAT (Fig 5, S7 Table).[40] The un-phased sites were enriched in cellular differentiation categories such as: *negative regulation of cell fate commitment*, but importantly a number of terms were related to inflammatory response (*regulation of cytokine production*, *regulation of interleukin-6 production*, *regulation of TNF production*). GO terms for phased sites were enriched in cellular and developmental terms (*skeletal system morphogenesis*, *response to organophosphorous*, *regulation of cell migration*, *cell-matrix adhesion*, *and osteoblast development)* In addition, binomial fold changes for un-phased AHR-TCF sites were ~10 times higher than for phased sites, implicating the indirect interaction of AHR-TCF21 factors as predominant in the human genome.

### In vivo co-localizing TCF21 and AHR/ARNT binding sites enrich for coronary artery and chronic inflammatory disease GWAS variants

**Fig 6:**
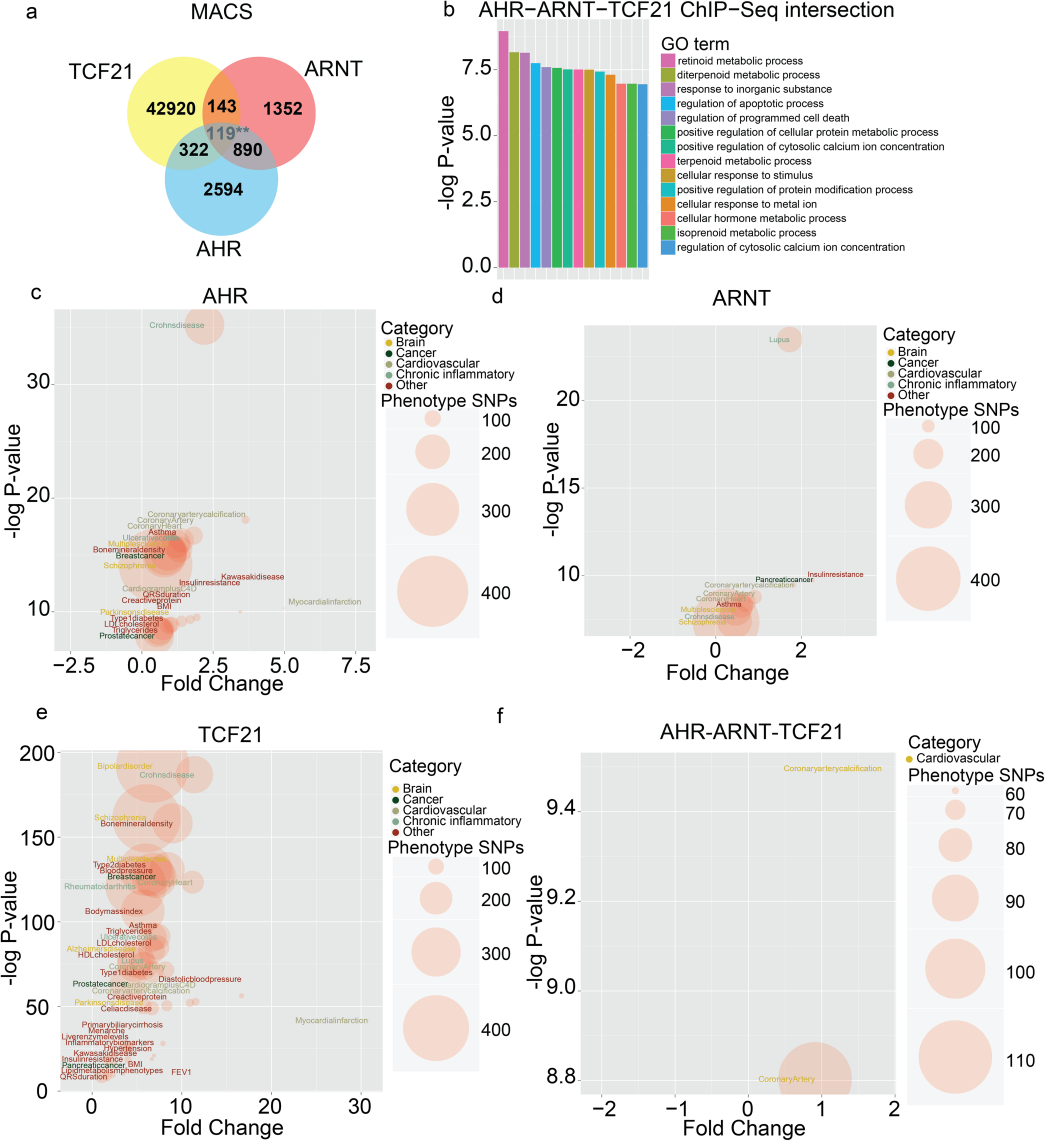
ChIP-seq data show co-localization of TCF21, AHR and ARNT binding in loci containing genes related to inflammation and programmed cell death. (a) Venn diagram showing intersection of the TCF21, AHR and ARNT ChIP-seq peaks. Note that the shown number of overlapping AHR-ARNT-TCF21 sites is colored in light gray to indicate that it is a subgroup of the shown intersections of individual pairs. (b) GO terms related to the overlap of TCF21, AHR and ARNT ChIP-seq sites. GO terms were defined using 119 overlapping sites from a, and by identification of related genes with GREAT. Calcium signaling GO terms are noted to represent a broad array of cytokines and chemokines in addition to other types of molecules. (c) Overlap of AHR ChIP-seq binding sites and GWAS SNPs in a window of +/-2000bp. GWAS SNPs were obtained from the GWAS Catalog, parsed into categories and the binomial enrichment of overlap with the AHR ChIP-seq sites was calculated for each parsed category. Plotted are binomial fold change vs.–log binomial p-value and terms were colored according to the four major categories: brain, cancer, cardiovascular, chronic inflammatory. (d) Overlap of ARNT ChIP-seq binding sites and GWAS SNPs in a window of +/-2000bp. (e) Overlap of TCF21 ChIP-seq binding sites and GWAS SNPs in a window of +/-2000bp. (f) Overlap of AHR-ARNT-TCF21 ChIP-seq binding sites and GWAS. SNPs in a window of +/-2000bp.

To further evaluate whether TCF21 and AHR-ARNT complex binding co-localizes in the human genome, we compared ChIP-seq data for these three TFs. We identified TCF21 ChIP-seq peaks in HCASMC that overlapped with AHR and ARNT ChIP-seq sites identified in MCF-7 cells[41], and obtained a statistically significant co-localization of sites using the Fisher’s exact test. Overlap of TCF21 and AHR peaks produced odds ratios within confidence intervals CI: 4.34–5.4 (p = 1.88e-121), for ARNT and TCF21, CI: 4.16–5.77 (p = 1.7e-56) and for AHR, ANRT and TCF21, CI: 4.91–7.05 (p = 1.13e-56) (S8 Table). We obtained in total 322 (12.4%) genomic locations that were co-occupied by AHR and TCF21, out of which 119 sites were also occupied by ARNT. Similarly, in 143 genomic locations (10.5%) ARNT co-localized with TCF21, out of which 119 were occupied with its binding partner AHR (Fig 6A). Overlap of AHR and ARNT identified 890 sites (AHR, 34.3%; ARNT, 65.8% percent of total sites), consistent with the fact the two proteins are known binding partners. Subsequently, we selected genes that are proximal to the overlapping TCF21-AHR, TCF21-ARNT and TCF21-AHR-ARNT ChIP-seq sites, and performed GO enrichment analysis with GREAT (Fig 6B, S8 Table). TCF21 and AHR-ARNT overlapping sites classified into GO-terms related to chemokine and cytokine signaling (*positive regulation of cytosolic calcium ion concentration*, *regulation of cytosolic calcium ion concentration*), apoptosis (*regulation of apoptotic process*, *regulation of programmed cell death*), metabolic processes (*cellular hormone metabolic process*, *isoprenoid metabolic process*), and cellular signaling (*cellular response to stimulus*).

Next, we assessed the binding of AHR, ARNT and TCF21 near lead SNPs from the GWAS Catalog (version 2016-05-08), expanded by addition of CARDIoGRAM+C4D meta-analysis data[2, 42], using the binomial test for genomic overlap. We scanned the lead SNPs using windows of +/-2kb, +/-5kb, and +/-10kb near the ChIP-seq binding sites and calculated the significance using the gwasanalytics tool (Fig 6C–6F, S8 Fig). Using the +/- 2kb window, TCF21 binding shows general enrichment near a wide range of GWAS SNPs for cardio-metabolic phenotypes (*coronary heart disease*, *blood pressure and type 2 diabetes*) as well as chronic inflammatory diseases (*Crohns disease*, *multiple sclerosis*, and *rheumatoid arthritis*), as well as skeletal phenotypes (*bone mineral density*) and in certain neurological disorders (*bipolar disorder and schizophrenia*). ARNT binding was localized near GWAS SNPs for chronic inflammatory diseases (*lupus erythematosus* and *ulcerative colitis*) and *prostate cancer* GWAS SNPs. AHR binding co-localized with CAD variants (*coronary artery disease*, *coronary artery calcification*) as well as chronic inflammatory GWAS SNPs (e.g., *Crohn’s disease*). After intersection of AHR with ARNT and TCF21 the only remaining categories were *coronary artery disease* and *coronary artery calcification*, narrowing the importance of the interaction of AHR/ARNT and TCF21 factors to pathophysiological processes in cardiovascular disease. Furthermore, we surveyed the overlap of CARDIoGRAM+C4D GWAS SNPs and AHR-ARNT PWM to consider the potential role of AHR in other CAD associated genes. In total 456 ARNT-AHR sites overlapped with CARDIOGRAM+C4D SNPs (lead plus LD r2>0.8), comprising 0.27 permil (low stringency) and 0.38 permil (high stringency) of total ARNT-AHR PWMs. In comparison, there were only 7 and 5 HNF1A and HNF1B sites, comprising 0.12/0.10 permil of total HNF1A/B sites (p<0.005, comparison of AHR-ARNT and HNF using Z-score test for proportions, S9 Fig).

### TCF21 and AHR coordinately regulate downstream loci but do not show evidence of cooperative binding

**Fig 7:**
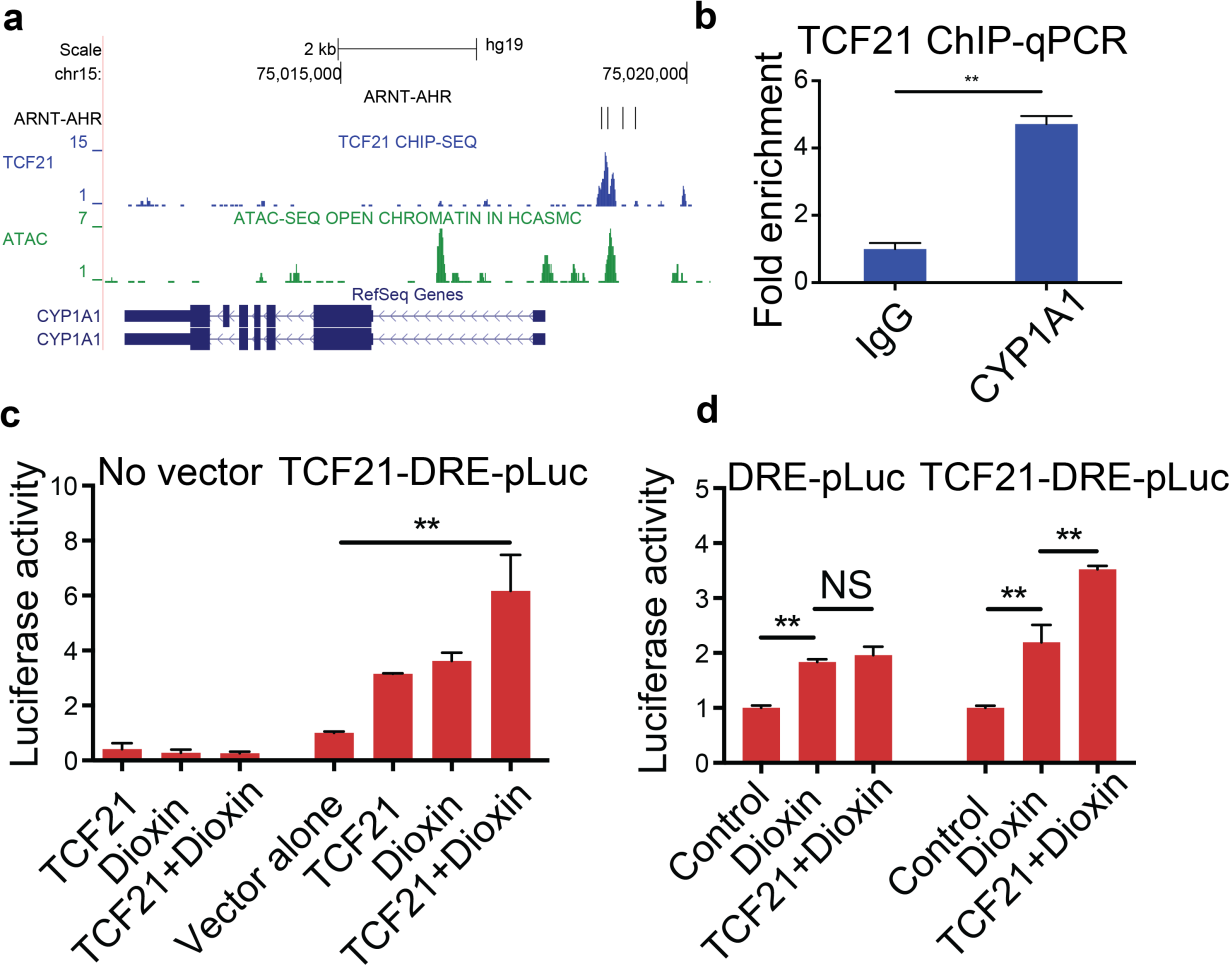
TCF21 binds in the *CYP1A1* locus, and interacts with AHR to promote transcription. (a) UCSC Genome Browser view of TCF21 binding upstream of the *CYP1A1* gene in a region of open chromatin as defined by ATAC-seq with a high predicted affinity for AHR-ARNT binding. (b) TCF21 binding to *CYP1A1* upstream region is demonstrated with ChIP-qPCR. (c) Transfection studies with a composite TCF21-AHR binding site reporter plasmid shows additive effects on transcription with combined *TCF21* expression plasmid and treatment of the cells with dioxin. *TCF21*-DRE-pLuc is a luciferase construct with alternating TCF21 and AHR binding motifs subcloned proximal to the promoter. DRE, dioxin response element. (d) Deletion of the TCF21 binding motif in the reporter plasmid abrogates the effects of *TCF21* over-expression, verifying that TCF21 has a direct transcriptional effect in these experiments. (*p<0.05; **p<0.005).

Given these data showing that TCF21 and AHR binding sites are co-localized in the genome (Figs 4–6), and that *TCF21* expression levels directly modulate the AHR response to dioxin (Fig 2), we investigated the functional interaction of these transcription factors at target loci. First, we surveyed the genomic region of *CYP1A1* for TCF21 in vivo binding in HCASMC. A TCF21 ChIP-seq binding peak was identified and localized to a region of open chromatin, as defined by ATAC-seq data in HCASMC[10], and binding was confirmed with ChIP-qPCR (IgG 1.0±0.18 vs. TCF21 4.71±0.24, p = 0.001) (Fig 7A and 7B). This peak co-localized in the same region of open chromatin with several predicted AHR-ARNT binding sites, thus suggesting coordinated regulation of *CYP1A1* expression. To confirm that the regulation of *CYP1A1* mRNA levels by AHR and TCF21 is mediated at the transcriptional level through the observed ChIP identified binding sites, and to look for evidence of cooperativity at this level, we conducted reporter gene transfection studies. A 50 bp sequence containing alternating binding motifs for TCF21 and AHR-ARNT binding identified in the *CYP1A1* gene was cloned into a luciferase expression plasmid with a minimal promoter sequence. Dual luciferase-renilla assays revealed enhancer activity when exposed to *TCF21* overexpression (1.0±0.06 vs. 3.15±0.02, p = 0.0024), and *TCF21* overexpression further increased the luciferase expression induced by dioxin in these cells (3.62±0.30 vs. 6.17±1.31 fold, p = 0.0005) (Fig 7C). The combined effect was additive with no evidence of synergism that would be suggestive of cooperative binding. Further, when the TCF21 binding motifs were removed from the reporter construct, *TCF21* overexpression failed to further increase the expression of luciferase, suggesting that the transcriptional effect of TCF21 is specific for protein-DNA binding (Fig 7D). These data indicate that the regulatory effect of TCF21 on AHR target genes can be mediated by direct interaction in these target loci, and requires protein-DNA binding.

### The *AHR* pathway is activated by dioxin and oxidized LDL in HCASMC

**Fig 8:**
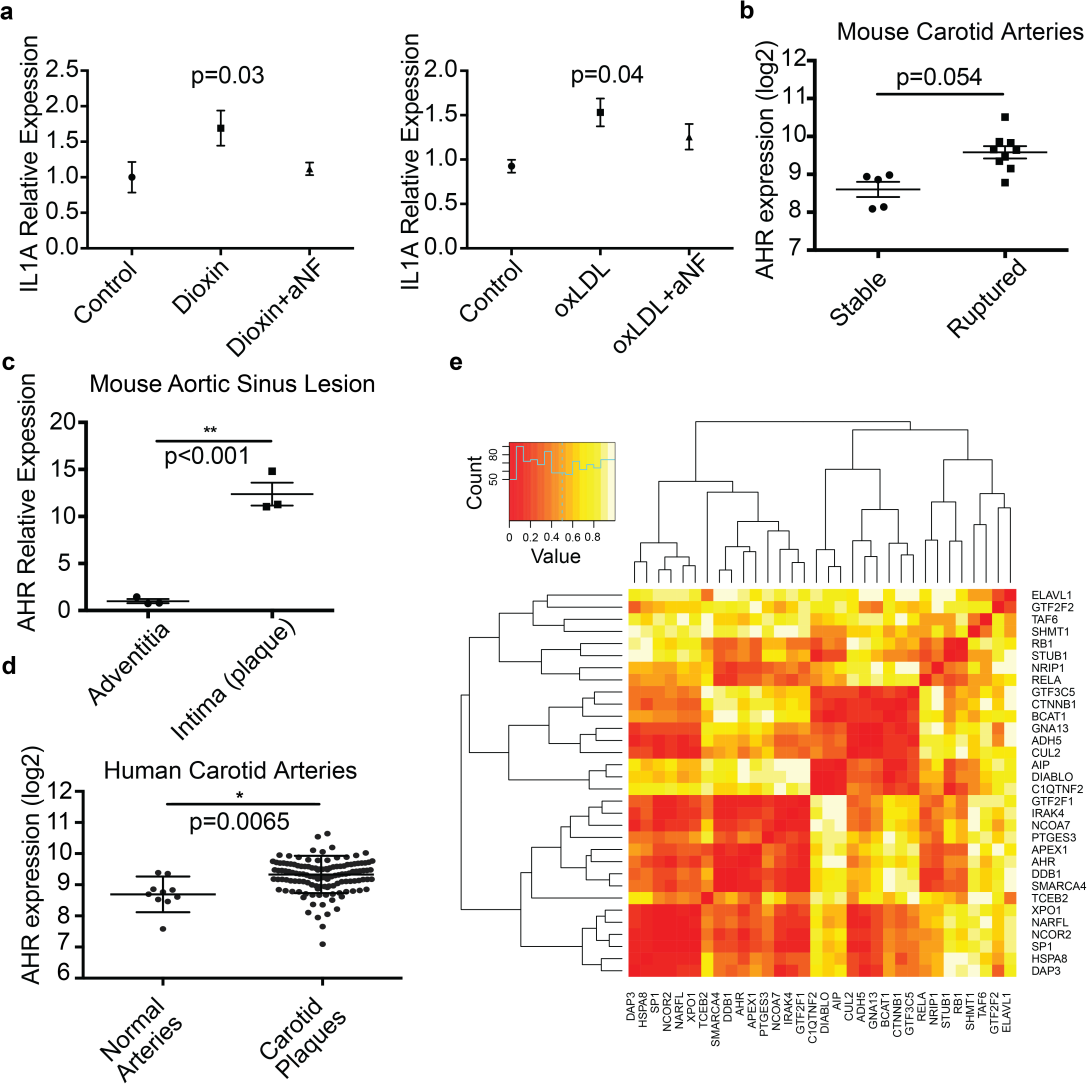
Endogenous regulation of the AHR pathway in SMC, and in vivo expression of *AHR* in atherosclerotic vascular disease. (a) *IL1A* mRNA levels are increased by dioxin and ox-LDL, and co-treatment with αNF, an AHR pathway antagonist, reverses these effects. (b) *AHR* mRNA levels are elevated in ligated carotid arteries with ruptured plaques compared to stable plaques. (c) Laser capture microdissection shows increased *AHR* mRNA levels to be located in the plaque region of the atherosclerotic aortic root. (d) *AHR* mRNA levels are elevated in atherosclerotic human carotid arteries compared to normal arteries. (e) In vivo protein expression patterns in human carotid artery plaque tissues. AHR-TCF21 unique interactors from the BIOGRID protein-protein interaction database display clustering patterns in mass-spectrometry and high resolution isoelectric focusing proteomic data from human carotid artery plaques. Proteomic datasets were constructed from patients with asymptomatic (10) and symptomatic (10) carotid stenoses. Mass-spec and high resolution isoelectric focusing yielded 8–9000 recovered proteins. Hierarchical clustering revealed AHR to be located in a cluster of genes that include immune related genes, transcription factors, and cell cycle regulated genes, such as IRAK4, SP1, and XPO1, respectively.

We followed up our previous studies showing regulation of inflammatory mediators by AHR and TCF21 with studies investigating possible endogenous mediators of AHR activation. As shown previously, application of dioxin to HCASMC resulted in up-regulation of *IL1A*, and this effect was reversed when cells were treated with the AHR antagonist alpha-napthoflavone (α-NF) (Fig 8A). In the same experiments, we tested oxidized-LDL (ox-LDL) as a potential endogenous activator of AHR in HCASMC.[43, 44] The treatment with ox-LDL resulted in the activation of genes that was similarly reduced with α-NF co-treatment, suggesting that the SMC response to ox-LDL is at least partly mediated by the AHR pathway (Fig 8A). We also found activation of a dioxin response element with oxLDL in luciferase assays (S10 Fig).

### *AHR* expression is increased in vivo in the atherosclerotic mouse aorta and in human diseased carotid arteries

Given these data suggesting that AHR targets overlap the TCF21 CAD associated transcriptional network genes, we sought to substantiate the relevance of *AHR* in vascular disease through expression studies in mouse and human vascular tissues[8–10] For in vivo gene expression in mice, we performed microarray analysis of carotid arteries subjected to plaque rupture induced by partial ligation in *ApoE*^*-/-*^ animals to compare gene expression between ruptured and non-ruptured plaques.[45] We found *Ahr* expression to be higher in the ruptured plaques compared to non-ruptured plaques (8.60±0.20 vs. 9.58±0.16, FDR q = 0.054) (Fig 8B). Furthermore, laser capture microdissection (LCM) was performed in atherosclerotic lesions in the aortic sinus of *ApoE*^*-/-*^ mice exposed to 12 weeks of high fat diet. We found the expression level of *Ahr* to be significantly higher in the intimal plaque when compared to the expression in the adventitia, localizing the expression of *AHR* to the pathologic intimal thickening (1.0±0.2 vs. 12.4±1.2, *p* = 0.0008) (Fig 8C). Next, we validated these findings in human arteries ex vivo, using microarray based expression data from normal arteries and atherosclerotic human carotid lesions from the BiKE repository.[46] Expression levels of *AHR* along with *IL1A*, and *MMP1* were significantly higher in the diseased lesions (Fig 8D, *AHR* 8.71±0.16 in normal vs. 9.27±0.05 in plaques, p = 0.0065; S11 Fig). Furthermore, we analyzed the proteins present in human carotid plaques using liquid chromatography tandem mass-spectrometry (LC-MS/MS). Proteomic datasets were constructed from highly phenotyped patients with asymptomatic and symptomatic carotid stenoses, 10 subjects each matched for gender, statin usage and age, with plaques selected on CT and histology criteria. AHR-TCF21 unique interactors from BIOGRID protein-protein interaction database were used to display clustering patterns (Fig 8E). AHR is located in a cluster of genes that include immune related genes such as IRAK4, interleukin-1 receptor-associated kinase 4, transcription factors like SP1, and cell cycle regulated genes including XPO1. In addition, we selected ChIP-seq co-occupied genes for AHR-TCF21 and AHR-ARNT-TCF21 transcription factors and observed clustering of AHR target protein CYP1B1 with extracellular matrix factors FN1, COL18A1 and with growth factor receptor IGF1R, implicating AHR and its downstream targets in regulation of extracellular matrix component of the diseased human carotid artery plaque (S12 Fig).

## Discussion

We have identified *TCF21* as the causal gene at 6q23.2, characterized its mechanism of association, and shown that binding of this transcription factor is enriched in other CAD associated loci.[8, 12] To investigate how TCF21 interaction with other CAD loci may regulate disease risk, we have begun to study mechanisms of association in these loci. For initial studies we have chosen the *AHR* gene, because it encodes a transcription factor, allowing direct study of its downstream signaling pathway, and because of the well-characterized link between this factor and environmental exposures that are relevant for cardiovascular disease. This work thus addresses two aspects of CAD that have not been directly approachable with human association studies, investigating both gene-by-gene and gene-by-environment contributions to disease genetic risk.

Although variants in the AHR locus (rs608646) have shown only nominal association with CAD risk in GWAS meta-analyses, this may be due to the technical limitation of the GWAS methodology in the AHR locus or inadequate statistical power. It remains possible if not likely that AHR functions as a hub or master regulator in CAD without harboring regulatory disease variants. We did identify a variant within the AHR locus (rs608646) that has a moderate association (p = 0.0047) in the CARDIOGRAM+C4D GWAS data (S1A Fig), and this SNP was also noted to regulate expression of AHR as identified with eQTL studies in GTEx tissues high in SMC content (S1B Fig). In aortic and coronary artery tissues, the AHR locus was enriched with AHR-eQTL signals with multiple peaks across the genomic region (Fig 3B, and S2 Fig), indicating that AHR gene expression is genetically regulated in the vasculature and emphasizing the relevance of AHR expression in these tissues. Further, we also found genome-wide enrichment of the PWM for AHR-ARNT within CARDIOGRAM+C4D GWAS loci (S9 Fig), suggesting that the effect of AHR on CAD may be partly via genetic variation in protein-DNA interaction near genes related to CAD. These data support the candidate gene study which found an association of CAD with the AHR locus in East Asians.[26]

In our studies, we have pursued numerous approaches to investigate links between these two genes and their related transcriptional networks, and to investigate mechanisms by which they may work together to modulate CAD risk. First, we have shown with targeted studies that TCF21 binds both the *AHR* and *ARNT* loci, and increases expression levels of these genes in HCASMC, confirming previously published genomic studies and RNA-seq studies reported here. Second, these studies provide evidence for overlap of the TCF21 and AHR transcriptional networks. Both TCF21 and dioxin were shown to increase expression of disease-related factors such as *IL1A*, *MMP1* and interestingly knockdown of *TCF21* was able to almost completely abolish the effect of dioxin, suggesting that the inflammatory activation by AHR is dependent on the presence of TCF21. AHR is well known to promote inflammation in a number of situations, and to work with NFkB in this regard.[17] Also, we have previously shown that TCF21 can promote expression of a number of inflammatory genes and we show that this pro-inflammatory program represents an intersection of TCF21 with AHR function, identifying a subset of TCF21 target genes that could create a highly inflammatory cellular profile that would be significantly magnified with relevant environmental exposures. We also found that oxidized LDL activated the AHR pathway in HCASMC, consistent with previous reports in other cell types.[43, 47]

Further analyses investigated additional mechanisms of interaction between these two pathways. Using PWMs for both TCF21 and AHR, we found highly significant enrichment for co-localization in regions of open chromatin in HCASMC, and characterized similar organization of these binding sites around transcription factor start sites, suggesting functional interaction between TCF21 and AHR and the basal transcriptional apparatus, as proposed previously for other TFs.[48, 49] The genomic co-localization was further refined by intersecting summit locations from TCF21 ChIP-seq data with AHR-ARNT PWM positions. Support for these observations reflecting in vivo associations was provided by co-localization of ChIP-seq peaks for TCF21, AHR, and ARNT. These data suggest a role for AHR-ARNT in the functional regulation of coronary SMC phenotype.

Co-localization of TF binding often suggests direct functional interaction, and since TCF21 and AHR may regulate transcription in the same direction, an obvious hypothesis is that they bind cooperatively either through direct protein-protein interaction or through joint recruitment of ancillary adaptor proteins.[50] In addition to the genomic co-localization data, the absence of *IL1A* response to dioxin in *TCF21* knockdown, and our studies investigating the phasing of binding site placement also suggests some form of direct or indirect molecular interaction. The striking difference between functional annotations for the two categories of steric relationship are consistent with different functional interactions between AHR–ARNT and TCF21 in the context of DNA binding. GO terms for un-phased sites showed much stronger enrichment and significance compared to those of the phased sites, supporting indirect interaction as the likely functional mechanism. We investigated this possibility using the *CYP1A1* gene as model locus where both transcription factors bind. The reporter gene transfection studies with constructs containing both TCF21 and AHR binding sites showed an additive effect suggesting that the transcriptional effects are due to each TF acting independently. However, both TCF21 and AHR are known to bind AR[51, 52], and also potentially bind to Rb1 (S13 Fig). It remains a possibility that they interact through other TFs that function as intermediaries and are either not expressed or not active in the HCASMC. In fact, we were not able to demonstrate direct protein-protein interaction using a co-immunoprecipitation assay. Future studies using chemical crosslinking with ChIP and serial ChIP may help resolve these important questions.

The functional role of TCF21 in vascular disease appears closely related to its role in embryonic coronary artery vascular development where it is expressed in SMC precursor cells, supporting proliferation and migration of these cells. AHR is also expressed in the developing coronary circulation, and the application of the AHR-ligand dioxin in zebrafish inhibited epicardial and proepicardial development.[53, 54] Both *TCF21* and *AHR* are downstream of retinoic acid signaling pathways that are critical in coronary artery development.[55–58] The possible molecular interaction of *TCF21* and *AHR* in this setting has not been established.

**Fig 9:**
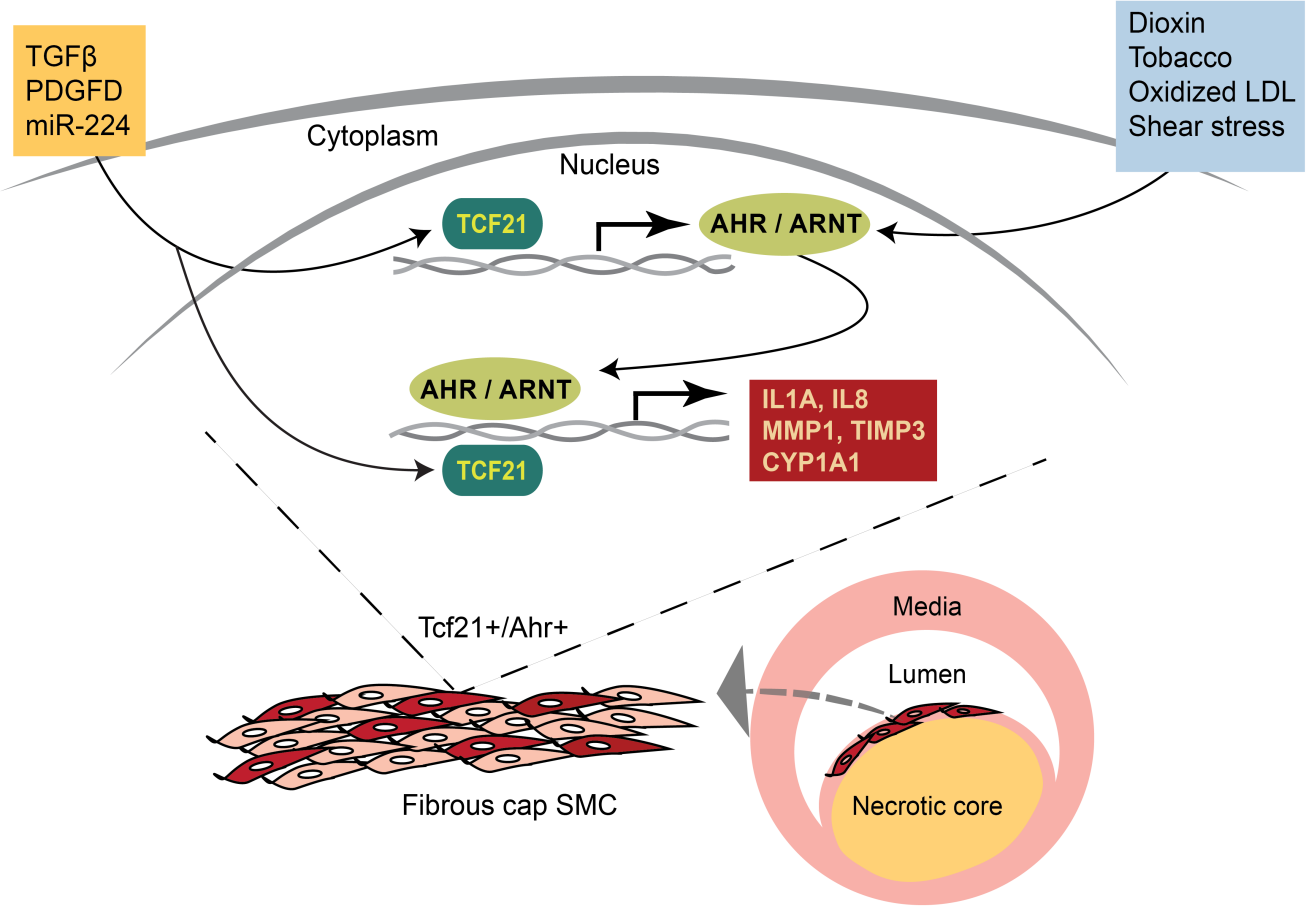
Schematic representation of AHR-TCF21 interactions that may modulate the effect of environmental stimuli on the progression of inflammation and atherosclerotic plaque formation. Environmental toxins, including dioxin and tobacco, as well as endogenous activators such as ox-LDL activate the AHR pathway, leading to increased inflammatory burden in the plaque. TCF21 can further increase the burden by increasing AHR expression as well as interacting with AHR at its downstream genes.

In summary, we describe a novel functional interaction between two bHLH class transcription factors and postulate association of their interaction to the development of atherosclerosis and coronary artery disease (Fig 9). The discovery of a connection between *TCF21*, one of the most highly replicated GWAS candidate genes for coronary artery disease, and *AHR*, a gene classically involved with response to environmental toxins raises an interesting hypothesis that this interaction may reflect gene-environment interactions that are contributing to CAD and presents an opportunity to define causal gene-gene and gene-environment interactions relevant to the atherosclerotic lesion. Furthermore, our findings that *TCF21* and *AHR* are expressed in the atherosclerotic plaque and that they interact to modulate inflammatory genes and matrix modifying genes suggest that the interaction may directly promote plaque instability leading to myocardial infarction. This work serves to promote mechanistic studies as an approach to understanding gene-by-gene and gene-by-environment contributions to disease genetic risk. Also, once the exact nature of the interaction of the two proteins is fully elucidated, therapeutic targeting of the pro-inflammatory interaction of TCF21 and AHR might be possible to reduce the effects of the pro-atherogenic stimuli in the vasculature and consequently reduce disease risk.

## Materials and methods

### Primary cell culture and reagents

Primary human coronary artery smooth muscle cells (HCASMC) were purchased from three different manufacturers, Lonza, PromoCell and Cell Applications and were cultured in complete smooth muscle basal media (Lonza, #CC-3182) according to the manufacturer's instructions. All experiments were performed with HCASMC between passages 5–8. HEK293 cells were maintained in DMEM containing high glucose, sodium pyruvate and L-glutamine supplemented with 10% FBS.

### TCF21 overexpression and knock-down

For the TCF21 overexpression study, HCASMC were transduced with 2^nd^ generation lentivirus with TCF21 cDNA cloned into pWPI (Addgene #12254). Briefly, for lentiviral transduction, the cells were treated at 60% confluence with MOI of 5 for 24 hours. The virus was removed and replaced with low-serum media for 48 hours prior to collection for downstream applications. For the siRNA transfection, cells were grown to 60% confluence, then treated with 10nM siRNA or scramble control with RNAiMax (Invitrogen, Carlsbad, CA) for 12 hours. The cells were collected 48 hours after transduction and processed using RNeasy kit (Qiagen, Hilden, Germany) for RNA isolation. *TCF21* knockdown was performed with siRNA oligos (Origene, Rockville, MD) using Lipofectamine RNAiMAX (Thermo Fisher Scientific, Waltham, MA) following manufacturer’s protocol.

### RNA isolation and quantification

RNA was isolated using RNeasy mini kit (Qiagen) and total cDNA was prepared using iScript cDNA synthesis kit (Biorad, Hercules, CA). Gene expression levels were measured using SYBR Green assays with custom designed probes (S9 Table) and quantified on a ViiA7 Real-Time PCR system (Applied Biosystems, Foster City, CA) and normalized to GAPDH levels. Two group comparisons were performed using student t-test, and three group comparison was performed with ANOVA.

### RNA-Seq and data analysis

HCASMC were cultured as described above and total RNA was purified from 5.0x10^5^ cells using the Qiagen miRNeasy kit. RNA libraries were prepared using the Illumina TruSeq library kit as described by the manufacturer. RNA molecules were sequenced using Illumina HiSeq 2500. Reads contained in raw *fastq* files were mapped to hg19 using the RNA-seq aligner STAR (v2.4.0i), that processes data with short run times and yields high numbers of uniquely mapped reads (https://github.com/alexdobin/STAR). Second pass mapping with STAR was then performed using a new index that is created with splice junction information contained in the file SJ.out.tab from the first pass STAR mapping. Read that have been mapped with STAR second pass mapping algorithm were subsequently counted using the htseq-count script distributed with the HTSeq Python package (https://pypi.python.org/pypi/HTSeq). Differential expression of exons, genes, and transcripts were assayed using the DESeq2 R package from Bioconductor (http://bioconductor.org/packages/release/bioc/html/DESeq2.html), which uses negative binomial distribution to estimate dispersion and model differential expression such as to permit biological variability to be different among tested genes (transcripts). GO terms enrichment and PCA analysis was performed using GOSeq and Gene Set Enrichment and Projection Displays–GSEPD Bioconductor package.

### HCASMC eQTL and allele-specific expression analysis

Fifty-two human coronary artery smooth muscle cell lines are genotyped using 30X whole-genome sequencing. Genotype calling follows the GATK best practices recommendations. Briefly, after removing adapter with cutadapt, trimmed FASTQ files were aligned with BWA mem, duplicates were marked with Picard tools. After indel realignment and variant base quality recalibration, single-nucleotide variants and short insertion and deletion variants are jointly called on all samples using the GATK Haplotype caller. Called variants are recalibrated and filtered using GATK's variant quality score recalibration module. We used BEAGLE 4.1 to impute and phase recalibrated variants using 1000 Genome phase 3 version 5a as a reference panel. After imputation and phasing, we filtered variants based on MAF > 0.05, Hardy-Weinberg equilibrium p-value > 1e-6, indel length < 51 bps, dosage r2 > 0.8. Gene expression was quantified using mRNA sequencing to an average depth of 50M 75-bp paired-ended reads. Sequences were aligned using STAR two-pass mapping. To avoid allele-specific mapping bias, we removed potentially mismapped reads using WASP. Read counts and FPKM values were generated using RNAseQC. Expression eQTL were mapped with RASQUAL. To remove potential confounders, we included gender, first 3 principal components inferred on the genotypes and first 8 PEER factors inferred on 10,000 highest expressed genes. Transcription factor binding and epigenetic annotations of variants were assayed by Haploreg v4.1.

### ChIP-qPCR

A detailed protocol was included in a previous publication [12]. HCASMC were cultured as described above. Antibodies used for ChIP-qPCR were all pre-validated according to ChIP-seq guidelines and ENCODE best practices. Purified rabbit polyclonal antibody against human TCF21 (HPA013189) was purchased from Sigma. Briefly, ChIP-qPCR confirmation was performed using primers designed for the genomic region of *AHR*, *ARNT* and *CYP1A1*, and compared against ChIP performed with IgG antibody. (S2 Table). qPCR values for *AHR*, *ANRT*, and *CYP1A1* promoter, normalized relative to the Myogenin (*MYOG*) signal, used as a endogenous control, were expressed as fold change compared to IgG ChIP sample. Comparisons were performed using student t-test.

### ChIP-Seq data analysis

TCF21 ChIP-Seq raw data from Sazonova et al., were reanalyzed using Model-based Analysis for ChIP-Seq (MACS v1.4.2) pipeline. Parameters were set to default. Summit locations of the peaks were defined for genome wide correlations with PWM using ChIPCor module—part of ChIP-Seq Analysis Server of the Swiss Institute of Bioinformatics (ccg.vital-it.ch/chipseq/chip_cor.php). TCF21 ChIP-Seq sites were converted to bigwig files and visualized on UCSC Genome Browser.

### *TCF21* and *AHR* interaction studies

*TCF21* overexpression was achieved using cDNA expression construct driven by a CMV promoter transduced by lentivirus. *TCF21* knockdown was performed with siRNA oligos (Origene) following manufacturer’s protocol. Briefly, for lentiviral transduction, the cells were treated at 60% confluence with MOI of 5 for 24 hours. The virus was removed and replaced with low-serum media for 48 hours prior to collection for downstream applications. For the siRNA transfection, cells were grown to 60% confluence, then treated with 10nM siRNA or scramble control with RNAiMax (Invitrogen) for 12 hours. The transduced cells were then treated with TCDD (Sigma Aldrich Cat#48599) at a concentration of 10nM for 24 hours. For the dual luciferase assay, double stranded DNA sequences containing the *TCF21* and *AHR* binding motifs were subcloned into the multiple cloning site (MCS) of the pLuc-MCS vector (Promega, #E1330), located upstream of the translation stop codon and firefly luciferase reporter gene *luc2*, driven by the PGK minimal promoter and also carrying the renilla luciferase reporter gene *hRluc*, as an internal control. Culture media was changed after 6 hrs, and dual luciferase activity was measured after 24 hrs using either SpectraMax L luminometer (Molecular Devices, Sunnyvale, CA). Relative luciferase activity (firefly/*Renilla* luciferase ratio) is represented as the fold change of respective control condition as indicated.

### Oxidized LDL treatment of HCASMC

Oxidized-LDL was purchased from Alfa Aesar (Haverhill, MA; Cat No. J65591). Cells were treated at concentration of 10uM for 6 hours with and without α-NF at 10nM (Sigma Aldrich, St. Louis, MO; Cat No. N5757). The changes in downstream genes were confirmed using RT-qPCR.

### Generation of mouse models for atherosclerosis of carotid artery and aortic sinus

12 week old *ApoE*^*-/-*^ mice on C56BL/6J background were subjected to 4 weeks of partial ligation followed by 4 days of cuff placement as described previously.[45, 59] For the aortic sinus atherosclerosis model, ApoE -/- mice were put on 12 weeks of Western high fat diet (HFD, 21% anhydrous milk fat, 19% casein and 0.15% cholesterol, Dyets no. 101511) at 4 weeks of age.

### RNA extraction from laser capture microdissection and cDNA amplification

Using a Leica LMD6000, we performed LCM of atherosclerotic plaques of mouse aortic sinuses. Briefly, following sacrifice, the cardiac chamber was perfused with PBS, then the aortic sinus was dissected and embedded in Optimal Cutting Temperature (OCT) medium (Tissue-Tek). 7um cryosections were placed on to Leica membrane slides, then visualized under the microscope for LCM. Total RNA was extracted using RNeasy Plus Micro kit (Qiagen), and the quality of RNA checked with Agilient Bioanalyzer RNA 6000 Pico kit. The levels of gene expressions were compared using total RNA generated from *ApoE* (-/-) mouse on high fat diet for 12 weeks. Using the published SMART-Seq2 protocol [60], we amplified the ultra-low input RNA from the LCM. Briefly, reverse transcription was performed using a template switching oligonucleotide (TSO) with locked nucleic acid (LNA) and Superscript II (Invitrogen, Carlsbad, CA), followed by PCR amplification with KAPA PCR polymerase.

### Microarray gene expression in human atherosclerotic carotid arteries

Human atherosclerotic carotid artery lesions were obtained from patients undergoing endarterectomy surgery for carotid stenosis, as part of the Biobank of Karolinska Endarterectomies (BiKE).[46] Details of the cohort demographics, sampling at surgery, processing and microarray analyses have been described before. Briefly, normal control samples (n = 10) were iliac arteries and one aorta from healthy organ donors without any history of cardiovascular disease. Plaques were frozen at **-**80**°**C immediately after surgery, pulverized to a powder before resuspending in Qiazol lysis reagent (Qiagen) and homogenization with a tissue homogenizer. Total RNA was extracted as described above using the miRNeasy Mini Kit (Qiagen) and RNA quality assessed using a Bioanalyzer 2100 (Agilent). Global gene expression profiles were analyzed by Affymetrix HG-U133 plus 2.0 Genechip microarrays from 127 patient derived plaque samples and 10 donor control samples. Robust multi-array average (RMA) normalization was performed and processed gene expression data presented in Log2 scale.

### LC-MS/MS analysis and protein identification

Atherosclerotic plaques from 18 BiKE patients (matched for male gender, age and statin medication) were analysed using LC-MS/MS as previously described.[46, 61] Briefly, protein samples were digested by trypsin and the resulting tryptic peptides were TMT-labeled and pooled. Pooled samples were cleaned by Strong Cation exchange columns (Phenomenex) and subjected to LC-MS/MS analysis. The sample pools were separated on a 4 hour gradient using an UPLC-system (Dionex UltiMate™ 3000) coupled to a Q-Exactive mass spectrometer (Thermo Fischer Scientific, San Jose, CA, USA). The fragment spectra from the mass spectrometer were matched to a database consisting of theoretical fragment spectra from all human proteins and filtered at a 1% False Discovery Rate (FDR) on the peptide level to obtain protein identities (Uniprot). Quantitative information was acquired by using the TMT reporter ion intensities. Correlation matrices were constructed by calculation of the proteomic expression correlation coefficients using the Pearson method and p-values were corrected for multiple comparisons using Bonferroni. For the clustering plots, dissimilarity index was created using the method that best discriminates all correlated pairs, given the formula: Dissimilarity = 1 –Abs (Correlation). Distance matrix was then created from the dissimilarity index. Clustering was performed with heatmap.2 in gplots.

### Co-expression modules

*AHR*, *ARNT*, *TCF21*, *HNF1A*, and *HNF1B* co-expression modules were obtained using GeneFriends using 4164 human microarray data sets or 4133 human RNA-Seq data sets. *TCF21* and *AHR* co-expression modules were defined using 4164 human microarray datasets through GeneFriends and visualized with Cytoscape. *TCF21*, *AHR* and *ARNT* co-expression modules were defined using 4133 human RNA-seq datasets through GeneFriends and visualized with Cytoscape. GeneFriends associations are defined with the threshold of 5%, meaning the gene is associated to a specific gene if it is in the top 5% co-expressed genes for a that gene. Cytoscape network file was imported for visualization from GeneFriends, containing all gene-gene associations marked as "good friends" (top 10 friends with a connection strength of 1), or "lesser friends" (genes ranking between 10 and 20 with a rank of 0.5). If a gene is an indirect connection, i.e. friend of a friend, score of 0.25 is deduced from the connection strength. Core network of direct interactions is marked on a graph with different colors to distinguish direct from indirect interactions. Connecting co-expression modules and visualization was performed using Cytoscape. Clustering was performed using Edge-weighted Spring Embedded layout. Coronary artery disease (CAD) GWAS genes were defined using Cardiogram plus C4D meta-analyses GWAS loci. In total, 77 CAD GWAS genes were used for transcription module analysis in GeneFriends:Microarray to obtain gene expression modules which were subsequently clustered in Cytoskape. Genes TCF21, AHR, COL4A1, SMAD3 and PDFGD from the main cluster were colored and indicated, node size was increased and edges to their first neighbors were colored in red. Underlying edge connections were colored purple with increased transparency. Grouping of transcription modules into three main clusters shows that CAD GWAS genes act through three main regulatory networks with TCF21 and AHR gene modules appearing in the single cluster.

### Position weight matrix analysis

TCF12 and AHR-ARNT matrices (TCF12—MA0521.1 and AHR-ARNT—MA0006.1) were obtained from the JASPAR database (http://jaspar.genereg.net) [35]. TCF12 PWM was used as it has the same binding motif as TCF21. Human genome hg19 was scanned with the two JASPAR matrices using PWMScan—Genome-wide PWM scanner (http://ccg.vital-it.ch/pwmtools/pwmscan.php). Position weight matrix sites were counted in windows of various lengths surrounding centered features using the Feature correlation tool from the ChIPCor module from ChIP-Seq Analysis Server (ccg.vital-it.ch/chipseq/chip_cor.php).

### GWAS binomial enrichment

AHR, ARNT and TCF21 ChIP-Seq binding site were extended to windows +/-1000bp, +/-2000bp, and +/-5000bp using *bedtools* package. Overlap of extended locations of AHR, ARNT and TCF21 ChIP-Seq binding sites and GWAS Catalog SNPs was performed with *bed2GwasCatalogBinomialMod1Ggplot* script from *gwasanalytics* package. This script is a modification of the *bed2GwasCatalogBinomialGgplot* and calculates binomial p-value for genomics overlaps using the following criteria. The P-values were computed using binomial cumulative distribution function b(x;n,p) in R (dbinom function). We set the parameter n equal to the total number of GWAS SNPs in a particular GWAS phenotype. Parameter x was set to the number of GWAS SNPs for a given GWAS phenotype that overlap input regions and parameter p was set to the fraction of the uniquely mappable human hg19 genome (calculated with subscript) that is localized in the input regions and contains assessed GWAS phenotype SNPs. Calculated binomial p-value equals the probability of having x or more of the n test genomic regions in the open chromatin domain given that the probability of that occurring for a single GWAS genomic location is p. Plots were made using ggplot2 package and the wes anderson color palette in R (https://github.com/karthik/wesanderson).

### Statistical analysis

All experiments were performed by the investigators blinded to the treatments/conditions during the data collection and analysis, using at least two independent preparations and treatments/conditions in triplicate. R/Bioconductor or GraphPad Prism 6.0 was used for statistical analysis. For enrichment analyses, we used both Fisher’s exact test and the cumulative binomial distribution test, as indicated. For comparisons between two groups of equal sample size (and assuming equal variance), an unpaired two-tailed Student’s t-test was performed or in cases of unequal sample sizes or variance a Welch’s unequal variances t-test was performed, as indicated. P values <0.05 were considered statistically significant. For multiple comparison testing, one-way analysis of variance (ANOVA) accompanied by Tukey’s post hoc test were used as appropriate. All error bars represent standard error of the mean (SE).

### Ethics statement

The BiKE study is approved by the Ethical Committee of Northern Stockholm with following ethical permits: EPN DNr 95–276/277; DNr 02–146; DNr 02–147, DNr 2005/83-31; DNR 2009/512-31/2; DNR 2009/295-31/2; 2011/950-32; 2012/619-32 and 213/2137-32. The project is performed under the Swedish biobank regulations and prospective sampling is approved with informed consent procedure (DNr 2009/512-31/2). BiKE is registered at Socialstyrelsen (The National Board of Health and Welfare) and Biobank of Karolinska and approved by the Swedish Data Inspection Agency (approval date/number 2002-09-30 DNr 916–2002). All samples are collected with oral and written informed consent from patients or organ donor guardians.

All animal procedures described in this study were approved by the Institutional Animal Care and Use Committees of Stanford University and conformed to NIH guidelines for care and use of laboratory animals. Specifically, the animal studies were approved by APLAC protocol #10022, last approved on 3-16-17 and will remain in effect until 12-12-19.

## Supporting information

S1 Table Analysis of differentially regulated genes in TCF21 overexpression with goseq. (XLSX)

S2 Table Analysis of differentially regulated genes in TCF21 overexpression with DAVID. (XLSX)

S3 Table Gene ontology analysis of significant associations between TCF21 and AHR networks. (XLSX)

S4 Table Analysis of rs10265174 correlation with AHR gene expression in GTEX database. (XLSX)

S5 Table Overlap of TCF21 and AHR-ARNT position weight matrices. (XLSX)

S6 Table TCF21 ChIP-Seq summits called with MACS. (XLSX)

S7 Table Separate GO enrichment analysis of genes near TCF21 and AHR-ARNT PWM overlap sites, where overlap is phased or un-phased. (XLSX)

S8 Table GO enrichment analysis of genes near TCF21, AHR and ARNT ChIP-Seq peaks. (XLSX)

S9 Table List of probes and primers used for experiments. (DOCX)

S1 Fig CAD GWAS and eQTL signals in the AHR locus. (PDF)

S2 Fig Active regulation of the AHR gene in aortic and coronary artery tissues. (PDF)

S3 Fig GO terms identified with TCF21 overexpression in HCASMC. (PDF)

S4 Fig TCF21, AHR, and ARNT co-expression modules build a strong interconnected network of genes. (PDF)

S5 Fig AHR and ARNT are upregulated by TCF21 overexpression. (PDF)

S6 Fig TCF21 binds AHR dimerization partner ARNT gene upstream region in human coronary artery smooth muscle cells. (PDF)

S7 Fig Control PWM correlations show absence of colocalization genome-wide. (PDF)

S8 Fig AHR-ARNT, AHR-TCF21 and AHR-ARNT-TCF21 overlapping ChIP-Seq sites enrich in coronary and inflammatory GWAS lead SNPs in window of +/-10kb and +/-5kb. (PDF)

S9 Fig Enrichment of AHR-ARNT PWM to SNPs in LD with CARDIOGRAM+C4D GWAS lead SNPs. (PDF)

S10 Fig Oxidized LDL is an activator of the AHR pathway. (PDF)

S11 Fig Increased expression of IL1A and MMP1 in human carotid artery plaques. (PDF)

S12 Fig AHR-TCF21 ChIP-Seq co-occupied genes display clustering patterns in mass-spectrometry and high resolution isoelectric focusing proteomic data from human carotid artery plaques. (PDF)

S13 Fig Network interactions of TCF21 and AHR target genes. (PDF)
