# Supplementary material for: TCF21 and the environmental sensor aryl-hydrocarbon receptor cooperate to activate a pro-inflammatory gene expression program in coronary artery smooth muscle cells
Source: PLoS Genet. 2017 May 8;13(5):e1006750. doi: 10.1371/journal.pgen.1006750 (PMC5439967; doi:10.1371/journal.pgen.1006750)
Supplement: S9 Table — (DOCX) [file pgen.1006750.s009.docx]

**Supplemental Table 9.**

**qPCR primers**

Custom probes

| **Gene** | **Forward** | **Reverse** |
| --- | --- | --- |
| *TCF21* | TCCTGGCTAACGACAAATACGA | TTTCCCGGCCACCATAAAGG |
| *AHR* | AGTTATCCTGGCCTCCGTTT | TCAGTTCTTAGGCTCAGCGTC |
| *ARNT* | TGACTCCTGTTTTGAACCAGC | CTGCTCCGAAGTTTATCCACAT |
| *IL1A* | CCTGTAGTTTCCCAGAAGAA | ACTGCCCAAGATGAAGACCA |
| *MMP1* | GGGAGATCATCGGGACAACTC | GGGCCTGGTTGAAAAGCAT |
| *CYP1A1* | GAGGCCAGAAGAAACTCCGT | CCCAGCTCAGCTCAGTACCT |
| *GAPDH* | TGCACCACCAACTGCTTAGC | GGCATGGACTGTGGTCATGAG |

Taqman probes

| Gene | Probe |
| --- | --- |
| *TCF21* | Hs00162646_m1 (Thermo Fisher Scientific) |
| *IL1A* | Hs00174092_m1 (Thermo Fisher Scientific) |
| *GAPDH* | Hs02758991_g1 (Thermo Fisher Scientific) |

**ChIP-qPCR primers**

| **Gene** | **Forward** | **Reverse** |
| --- | --- | --- |
| *AHR* | ATAGTGCTGAGAAGCGGGTG | TCCAGTCCCTGTACCTGACC |
| *ARNT* | CAGCCAGTCCAGTGAAGGG | CCTCTTACGCAAGGAGGGC |
| *CYP1A1* | CTCTAGGGGGCAGAGGTCAG | CCGGTCCTTCTCACGCAAC |
| *MYOG* | CCTTGATGTGCAGCAACAGC | CCAACGCCACAGAAACCTG |
