# Supplementary material for: TCF21 and the environmental sensor aryl-hydrocarbon receptor cooperate to activate a pro-inflammatory gene expression program in coronary artery smooth muscle cells
Source: PLoS Genet. 2017 May 8;13(5):e1006750. doi: 10.1371/journal.pgen.1006750 (PMC5439967; doi:10.1371/journal.pgen.1006750)
Supplement: S1 Fig — (PDF) [file pgen.1006750.s010.pdf]

Fig. S1

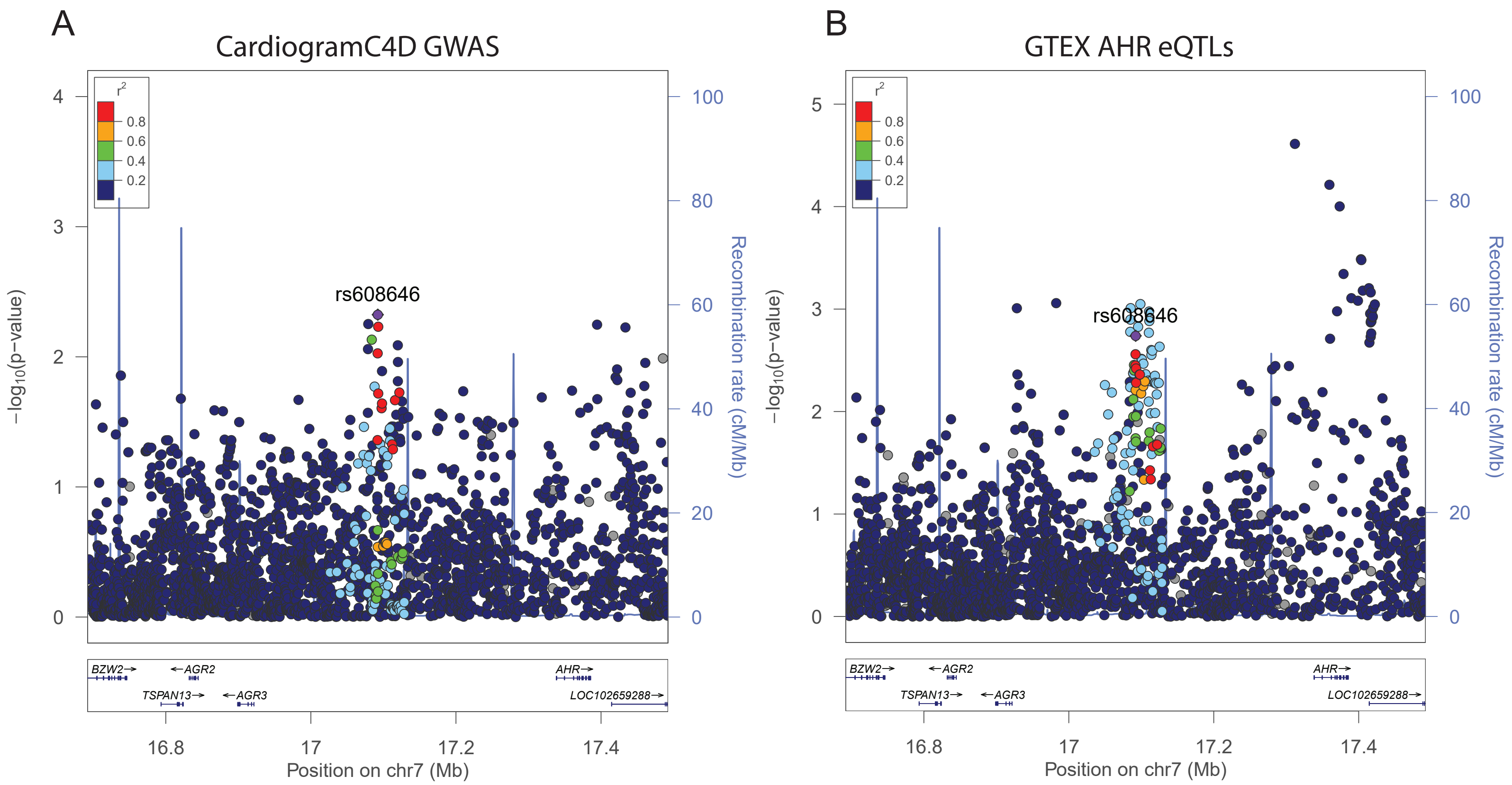

Figure S1. CAD GWAS and eQTL signals in the AHR locus

(A) Cardiogram+C4D association data at the AHR locus, showing nominal association of SNP rs608646 with the CAD phenotype. (B) AHR eQTL signal in esophagus mucosa for rs608646 identified in GTEx data. In addition, top significant GTEX tissues for rs608646 include smooth muscle cell enriched tissues such as esophagus muscularis, lung, colon transverse, and stomach.
