# Supplementary material for: TCF21 and the environmental sensor aryl-hydrocarbon receptor cooperate to activate a pro-inflammatory gene expression program in coronary artery smooth muscle cells
Source: PLoS Genet. 2017 May 8;13(5):e1006750. doi: 10.1371/journal.pgen.1006750 (PMC5439967; doi:10.1371/journal.pgen.1006750)
Supplement: S2 Fig — (PDF) [file pgen.1006750.s011.pdf]

Fig. S2

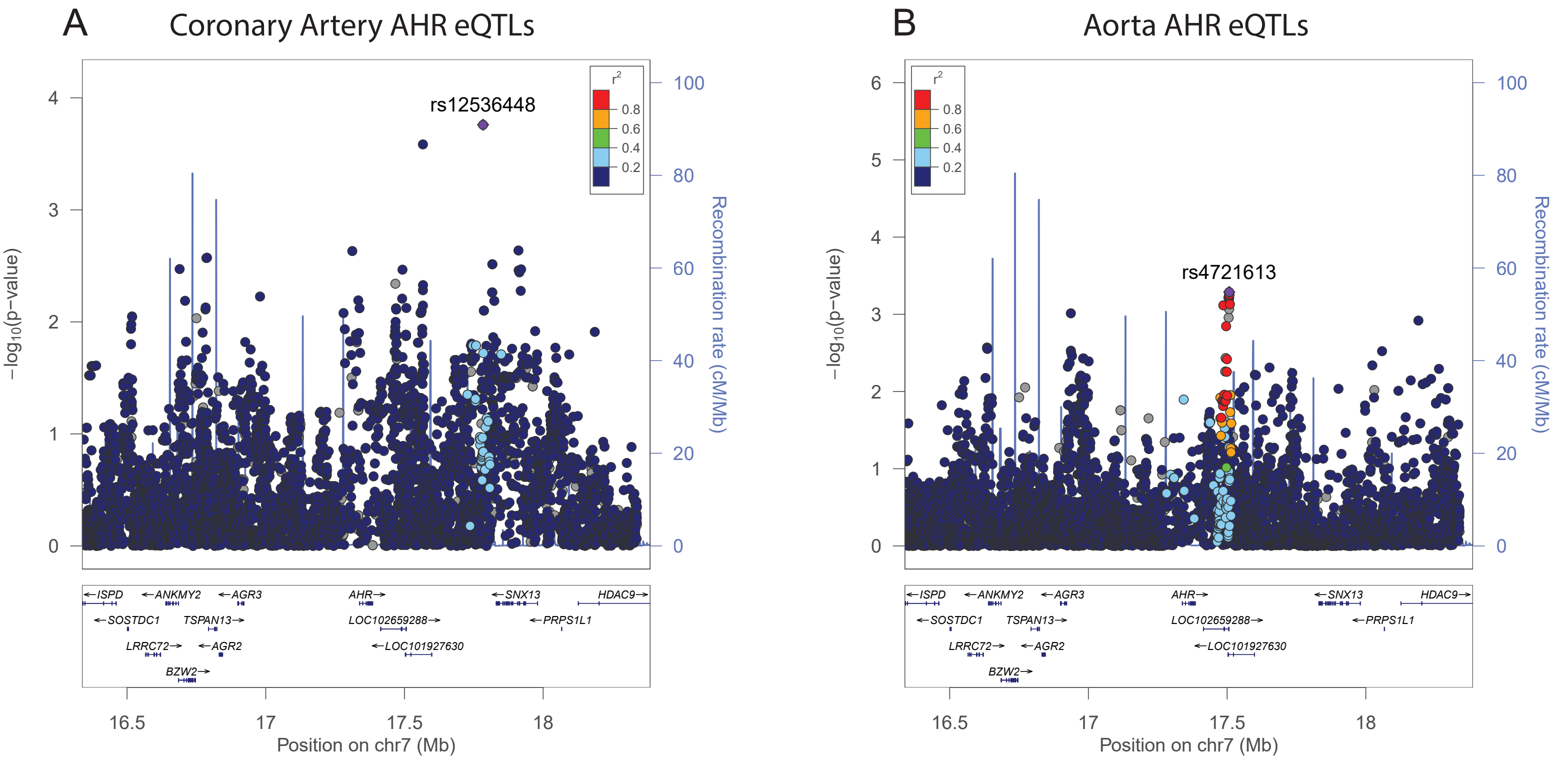

Figure S2. Active regulation of the AHR gene in aortic and coronary artery tissues.
(A) eQTL SNPs for the AHR gene from coronary artery tissues obtained from GTEX. (B) eQTL SNPs for the AHR gene from coronary artery tissues obtained from GTEX. The AHR locus shows active regulation of AHR gene expression by variants distributed throughout the locus with multiple regulatory regions that influence expression of AHR in these tissues.
