# Supplementary material for: TCF21 and the environmental sensor aryl-hydrocarbon receptor cooperate to activate a pro-inflammatory gene expression program in coronary artery smooth muscle cells
Source: PLoS Genet. 2017 May 8;13(5):e1006750. doi: 10.1371/journal.pgen.1006750 (PMC5439967; doi:10.1371/journal.pgen.1006750)
Supplement: S3 Fig — (PDF) [file pgen.1006750.s012.pdf]

A. Immune- related GO terms

Fig S3.

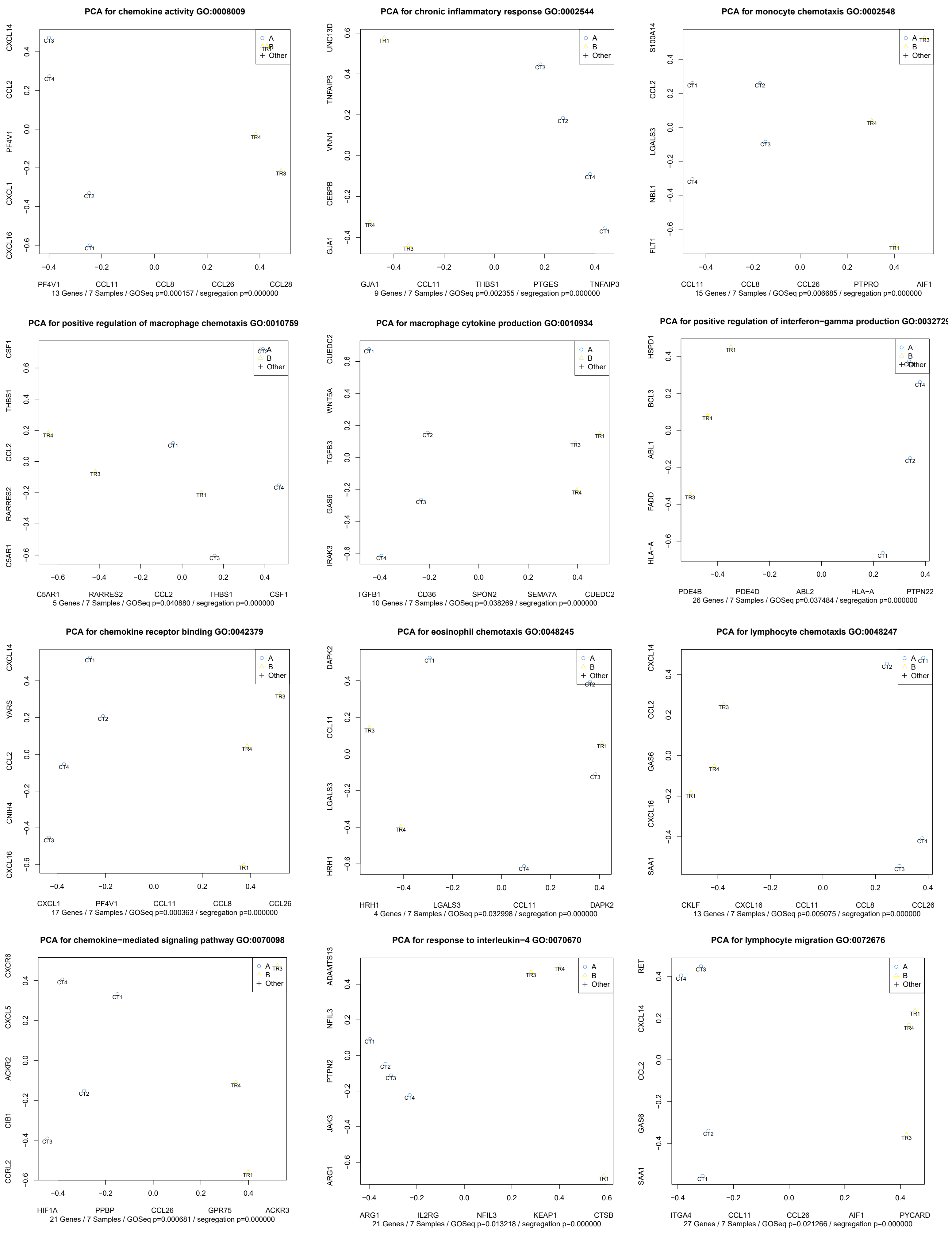

B. Atherosclerosis related GO terms

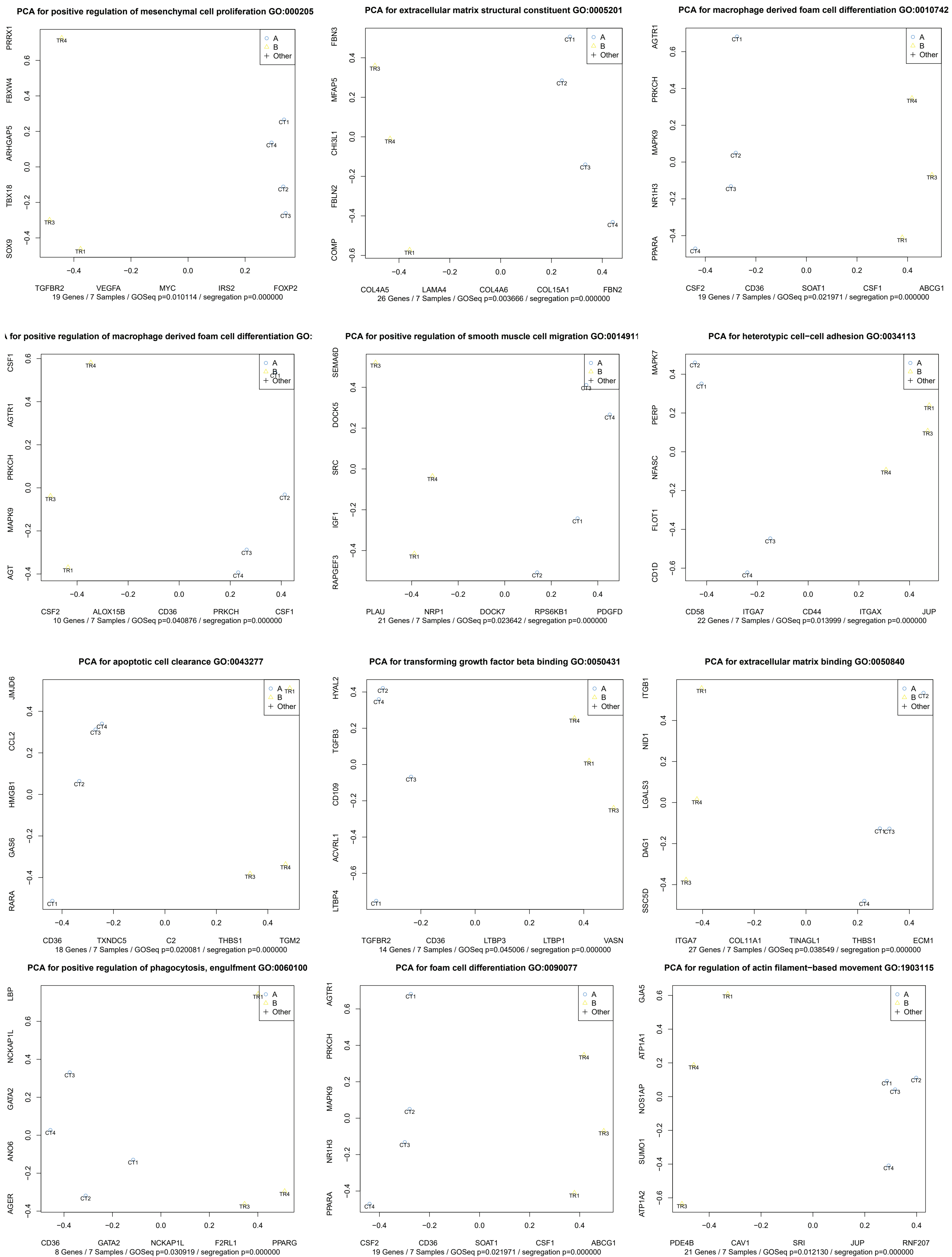

Figure S3.
GO terms identified with *TCF21* overexpression in HCASMC.
Differential expression of exons, genes, and transcripts were assayed using the DESeq2 R package from Bioconductor, which uses negative binomial distribution to estimate dispersion and model differential expression such as to permit biological variability to be different among tested genes (transcripts). GO terms enrichment and PCA analysis was performed using goseq and Gene Set Enrichment and Projection Displays – GSEPD Bioconductor package. Gene Ontology (GO) terms were grouped into following categories: A. Immune- related GO terms. B. Atherosclerosis- related terms.
