# Supplementary material for: TCF21 and the environmental sensor aryl-hydrocarbon receptor cooperate to activate a pro-inflammatory gene expression program in coronary artery smooth muscle cells
Source: PLoS Genet. 2017 May 8;13(5):e1006750. doi: 10.1371/journal.pgen.1006750 (PMC5439967; doi:10.1371/journal.pgen.1006750)
Supplement: S4 Fig — (PDF) [file pgen.1006750.s013.pdf]

Fig. S4

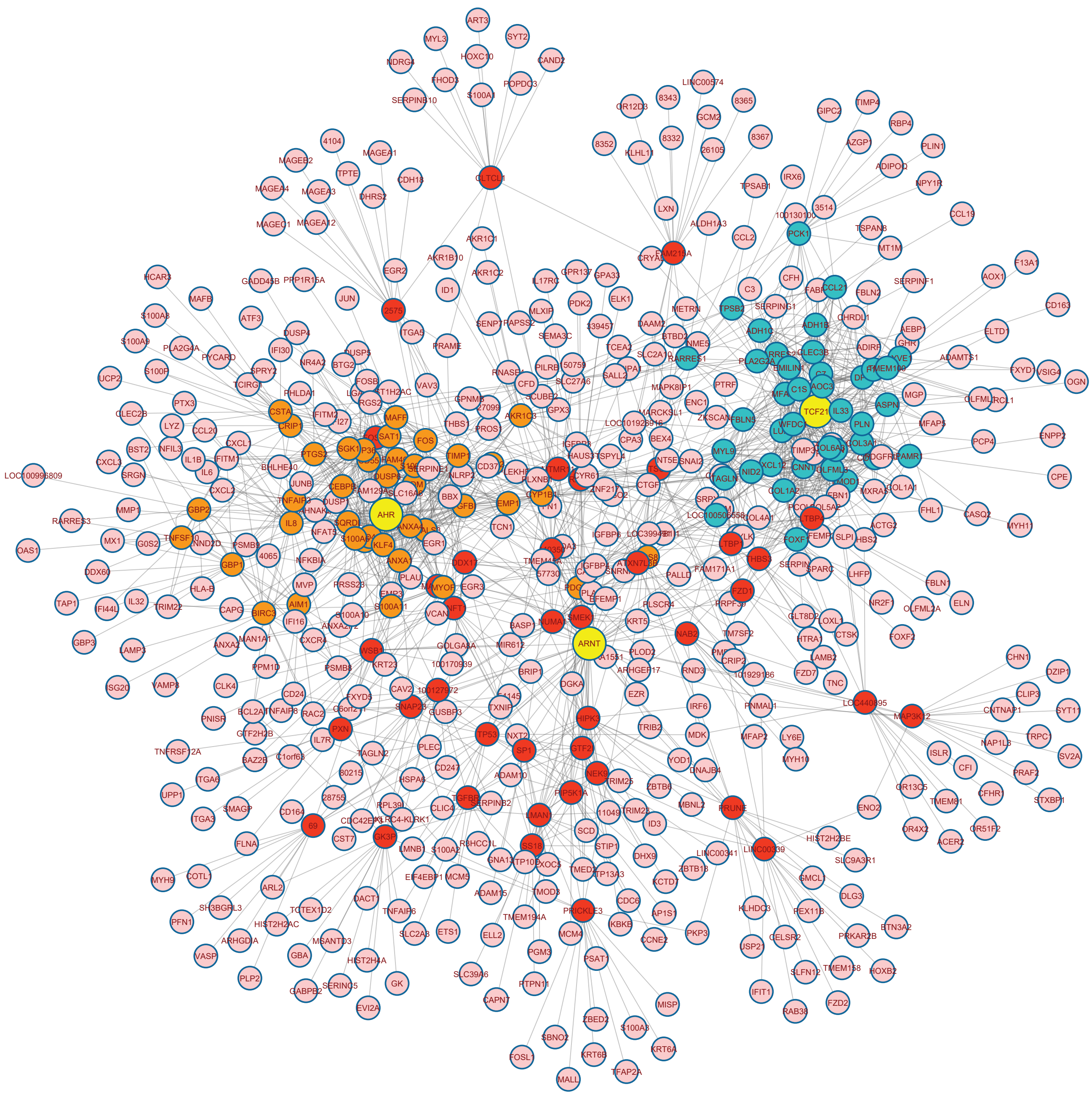

Figure S4. TCF21, AHR, and ARNT co-expression moduls build a strong interconnected network of genes

Co-expression modules of *TCF21* and *AHR-ARNT* show a high degree of connectivity. *TCF21, AHR* and *ARNT* co-expression modules were defined using 4133 human RNA-seq datasets through GeneFriends and visualized with Cytoscape. Colored in different colors for AHR, ARNT and TCF21 are genes that represent direct partners, i.e. genes that are in the top 5% co-expressed genes.
