# Supplementary material for: TCF21 and the environmental sensor aryl-hydrocarbon receptor cooperate to activate a pro-inflammatory gene expression program in coronary artery smooth muscle cells
Source: PLoS Genet. 2017 May 8;13(5):e1006750. doi: 10.1371/journal.pgen.1006750 (PMC5439967; doi:10.1371/journal.pgen.1006750)
Supplement: S5 Fig — (PDF) [file pgen.1006750.s014.pdf]

Fig. S5

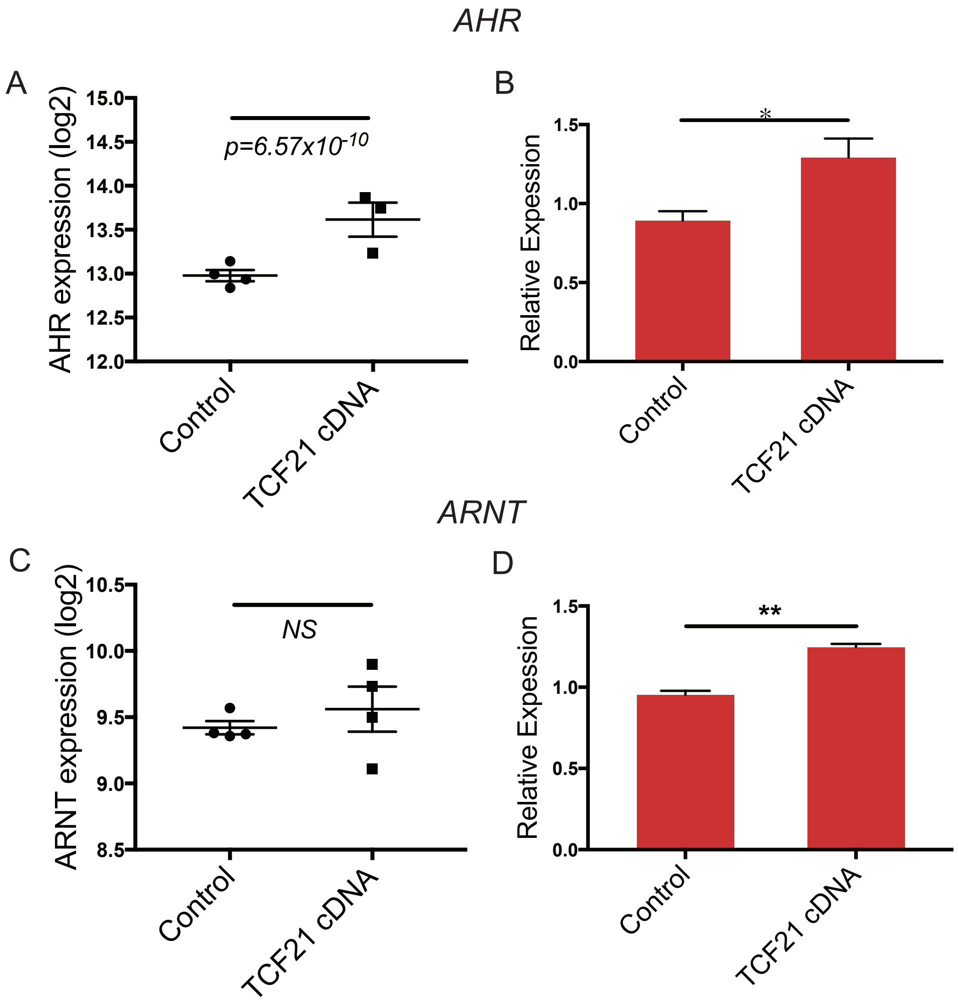

Figure S5. **AHR and ARNT are upregulated by TCF21 overexpression**
(**A**) RNA-Seq (FDR = 6.57x10-10), and (**B**) qPCR (0.89±0.06 vs. 1.29±0.12, p=0.018).
(**C**) ARNT is not significantly changed in TCF21 overexpression RNA-Seq, but (**D**) upregulation was confirmed with qPCR (0.95±0.02 vs. 1.25±0.02, p=0.0008).
