# Supplementary material for: TCF21 and the environmental sensor aryl-hydrocarbon receptor cooperate to activate a pro-inflammatory gene expression program in coronary artery smooth muscle cells
Source: PLoS Genet. 2017 May 8;13(5):e1006750. doi: 10.1371/journal.pgen.1006750 (PMC5439967; doi:10.1371/journal.pgen.1006750)
Supplement: S6 Fig — (PDF) [file pgen.1006750.s015.pdf]

Fig. S6

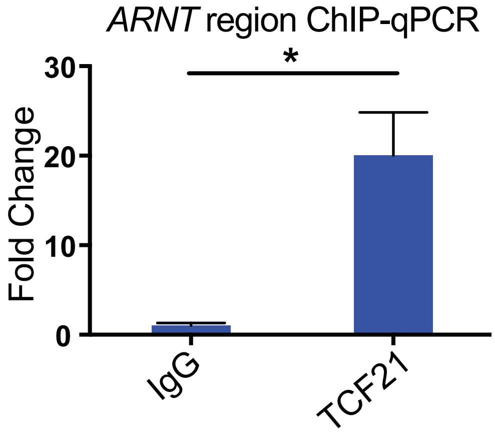

Figure S6. TCF21 binds AHR dimerization partner ARNT gene upsteream region in human coronary artery smooth muscle cells (HCASMC). Binding of TCF21 to ARNT upstream region in HCASMC validated using ChIP-qPCR compared to IgG control.
