# Supplementary material for: TCF21 and the environmental sensor aryl-hydrocarbon receptor cooperate to activate a pro-inflammatory gene expression program in coronary artery smooth muscle cells
Source: PLoS Genet. 2017 May 8;13(5):e1006750. doi: 10.1371/journal.pgen.1006750 (PMC5439967; doi:10.1371/journal.pgen.1006750)
Supplement: S7 Fig — (PDF) [file pgen.1006750.s016.pdf]

Fig. S7

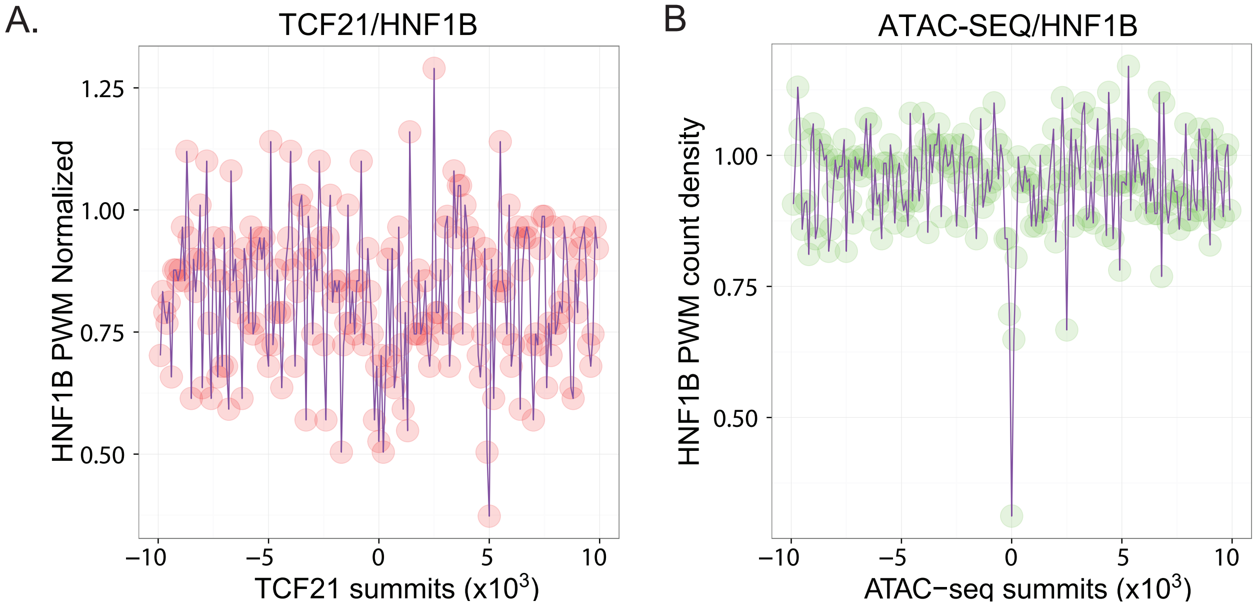

Figure S7. Control PWM correlations show absence of collocalization genome wide (A) Absence of genome wide correlation between negative control matrix HNF1B and TCF21 summits in HCASMC. Localization of HNF1A predicted binding sites and TCF21 ChIP-seq summits is shown as a density plot. Predicted sites were counted in a window of 100bp and normalized globally to the average number of HNF1B sites in 100bp windows. (B) Negative correlation between control HNF1B PWM and open chromatin in HCASMC. Localization of HNF1B predicted binding sites and open chromatin ATAC-seq summits is shown as a density plot.
