# Supplementary material for: TCF21 and the environmental sensor aryl-hydrocarbon receptor cooperate to activate a pro-inflammatory gene expression program in coronary artery smooth muscle cells
Source: PLoS Genet. 2017 May 8;13(5):e1006750. doi: 10.1371/journal.pgen.1006750 (PMC5439967; doi:10.1371/journal.pgen.1006750)
Supplement: S8 Fig — (PDF) [file pgen.1006750.s017.pdf]

Fig. S8

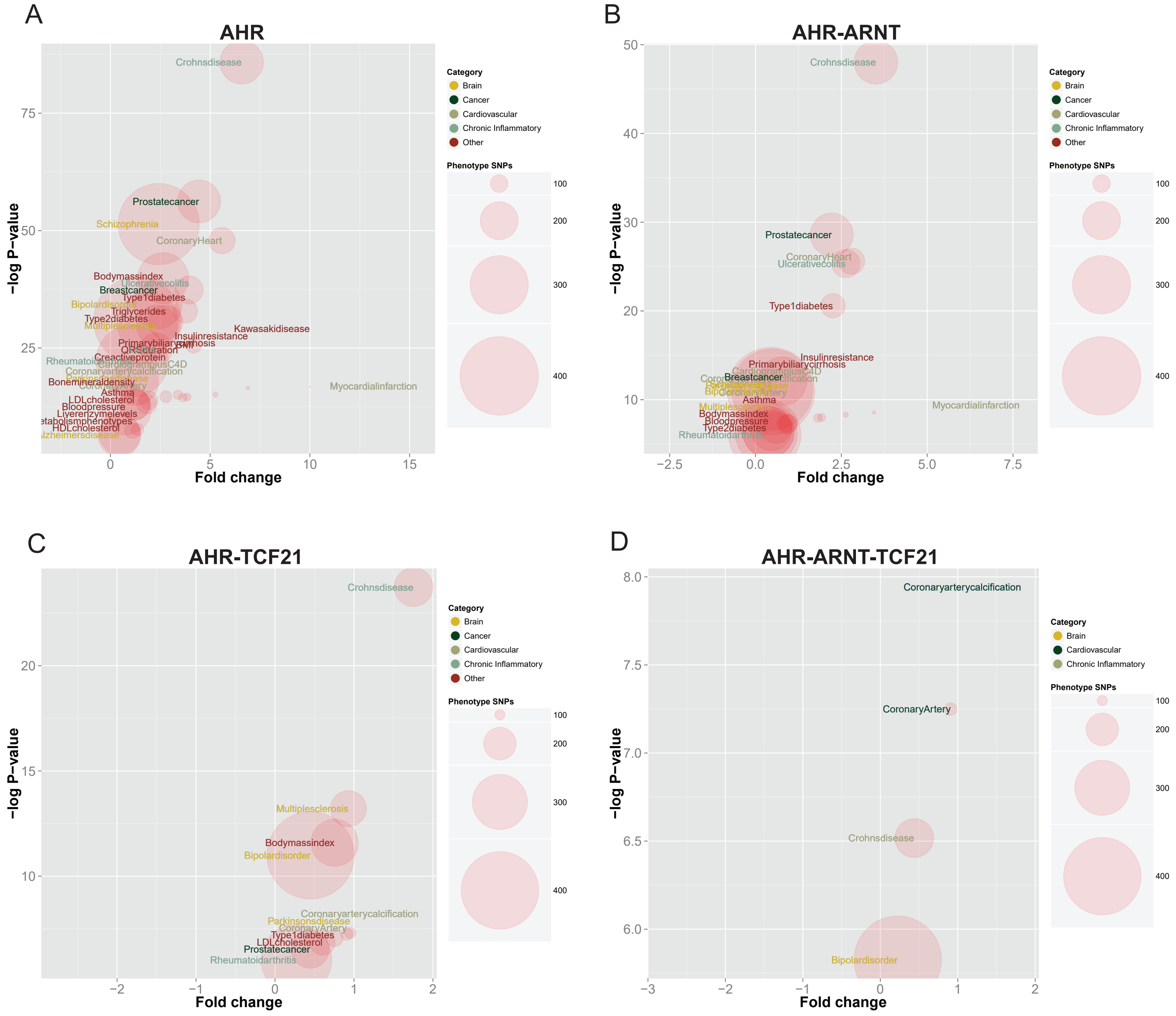
A
AHR
Crohnsdisease
Prostatecancer
Schizophrenia
CoronaryHeart
Bodymassindex
Ulcerativecolitis
Breastcancer
Type1diabetes
Bipolardisorder
Triglycerides
Type2diabetes
Multiplesclerosis
Kawasakidisease
Insulinresistance
Primarybiliarycirrhosis
QRSduration
Creactiveprotein
CardiogramplusC4D
Coronaryarterycalcification
Bonemineraldensity
Asthma
LDLcholesterol
Bloodpressure
Liverenzymelevels
HDLcholesterol
Myocardialinfarction
−log P−value
Fold change
Category
Brain
Cancer
Cardiovascular
Chronic Inflammatory
Other
Phenotype SNPs
100
200
300
400
B
AHR-ARNT
Crohnsdisease
Prostatecancer
CoronaryHeart
Ulcerativecolitis
Type1diabetes
Insulinresistance
Primarybiliarycirrhosis
CardiogramplusC4D
Breastcancer
Asthma
Bodymassindex
Bloodpressure
Type2diabetes
Rheumatoidarthritis
Myocardialinfarction
C
AHR-TCF21
Crohnsdisease
Multiplesclerosis
Bodymassindex
Bipolardisorder
Coronaryarterycalcification
Parkinsonsdisease
CoronaryArtery
Type1diabetes
LDLcholesterol
Prostatecancer
Rheumatoidarthritis
D
AHR-ARNT-TCF21
Coronaryarterycalcification
CoronaryArtery
Crohnsdisease
Bipolardisorder

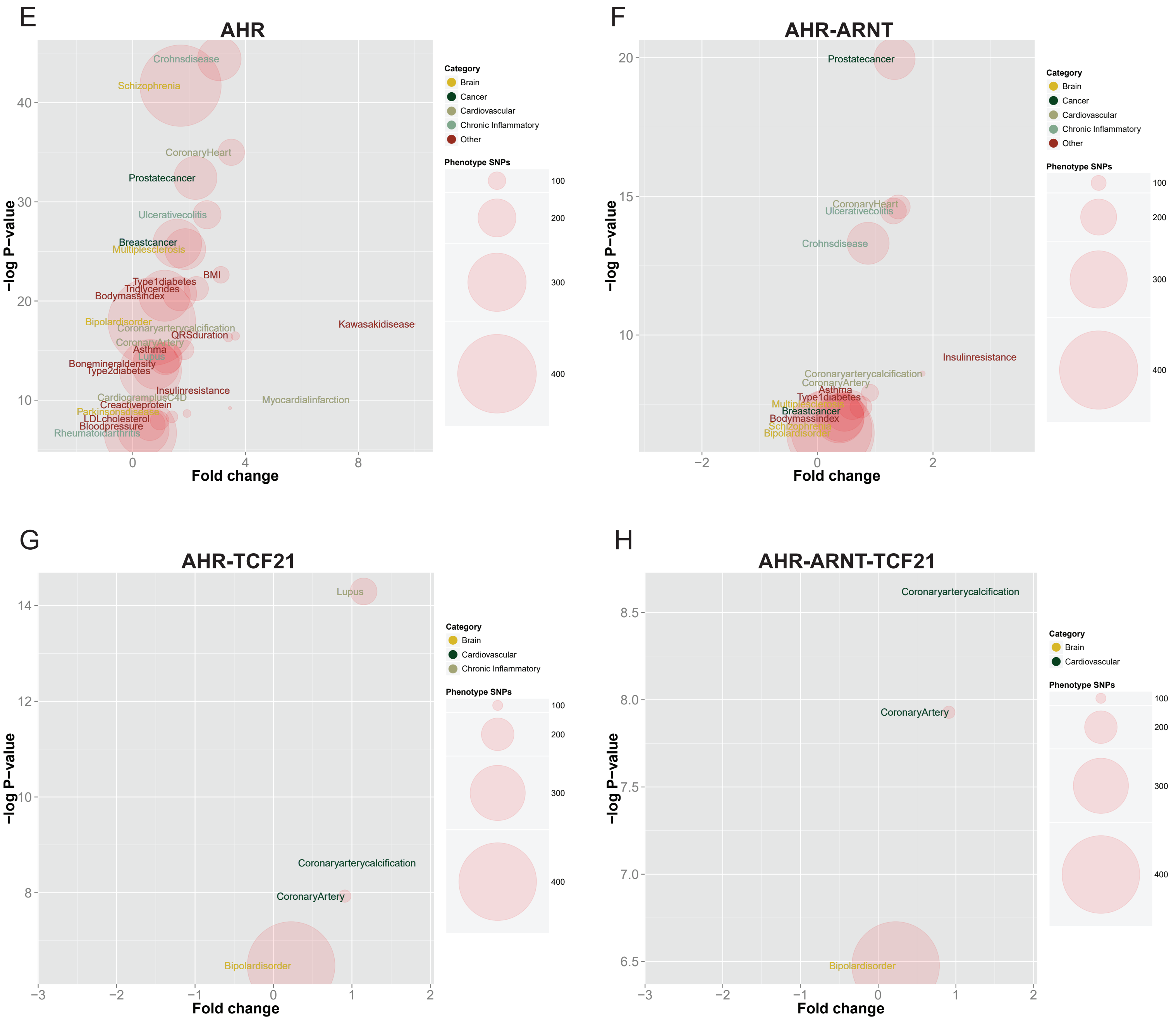

Figure S8.

AHR-ARNT, AHR-TCF21 and AHR-ARNT-TCF21 overlapping ChIP-Seq sites enrich in coronary and inflammatory GWAS lead SNPs in window of +/-10kb and +/-5kb

Overlap of (**A**) AHR ChIP-Seq binding sites, (**B**) AHR and ARNT ChIP-Seq binding sites, (**C**) AHR-TCF21 ChIP-Seq binding sites and (**D**) AHR-ARNT-TCF21 ChIP-Seq sites with GWAS SNPs in a window of +/-10000bp tested for significance using binomial test. Overlap of (**E**) AHR ChIP-Seq binding sites, (**F**) AHR and ARNT ChIP-Seq binding sites, (**G**) AHR-TCF21 ChIP-Seq binding sites and (**H**) AHR-ARNT-TCF21 ChIP-Seq sites with GWAS SNPs in a window of +/-5000bp tested for significance using binomial test.
