# Supplementary material for: TCF21 and the environmental sensor aryl-hydrocarbon receptor cooperate to activate a pro-inflammatory gene expression program in coronary artery smooth muscle cells
Source: PLoS Genet. 2017 May 8;13(5):e1006750. doi: 10.1371/journal.pgen.1006750 (PMC5439967; doi:10.1371/journal.pgen.1006750)
Supplement: S9 Fig — (PDF) [file pgen.1006750.s018.pdf]

Fig. S9

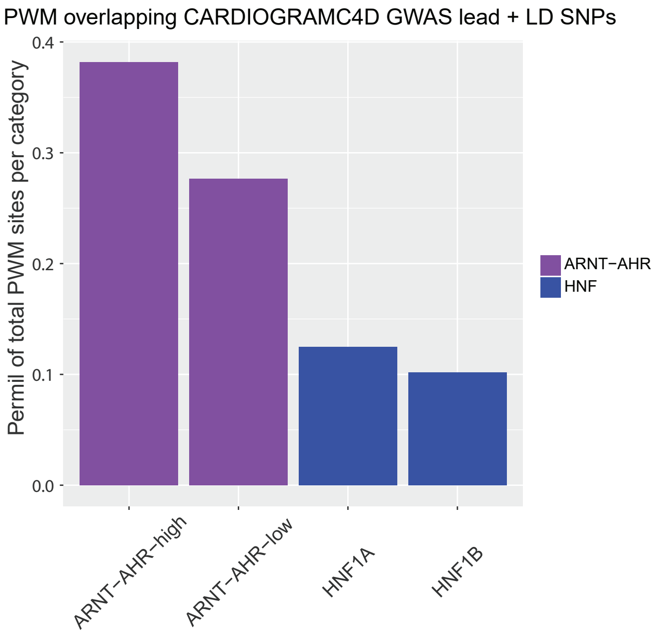

Figure S9. Enrichment of AHR-ARNT PWM to SNPs in LD with CARDIOGRAM+C4D GWAS lead SNPs.

Compared to control PWM, HNF1A and HNF1B, significantly higher proportion of PWM binding sites for AHR-ARNT co-localized to CARDIOGRAM+C4D GWAS lead SNPs plus SNPs in LD defined wirh r2>0.8 (statistical test, Z score=3.04, $p<0.005$). Similar trend was observed for both, low and high stringency cut-offs for ARNT-AHR PWM matrix. In total, 456 ARNT-AHR sites overlap CARDIOGRAM SNPs (lead plus LD D'>0.8).
