# Supplementary material for: TCF21 and the environmental sensor aryl-hydrocarbon receptor cooperate to activate a pro-inflammatory gene expression program in coronary artery smooth muscle cells
Source: PLoS Genet. 2017 May 8;13(5):e1006750. doi: 10.1371/journal.pgen.1006750 (PMC5439967; doi:10.1371/journal.pgen.1006750)
Supplement: S10 Fig — (PDF) [file pgen.1006750.s019.pdf]

Fig. S10

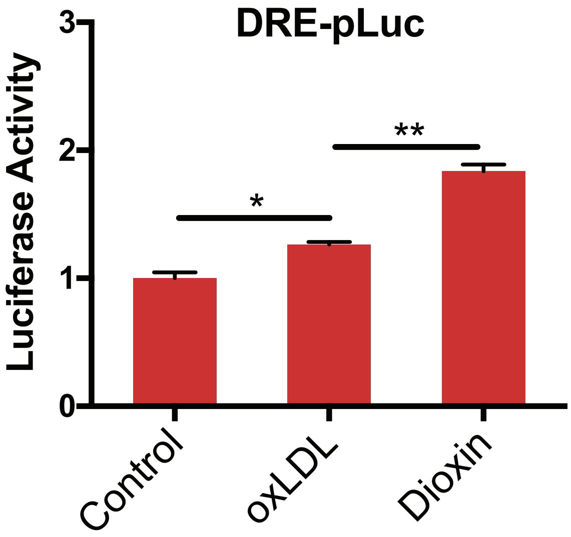

Figure S10. Oxidized LDL is an agonist of the AHR pathway

Luciferase reporter assay with dioxin response element (DRE) and minimal promoter driving the expression of the luciferase gene (pLuc). Both treatment with oxLDL (1.0±0.05 vs. 1.26±0.02; $p=0.01$) and Dioxin (1.0±0.05 vs.1.84±0.05; $p<0.001$) showed significant increase in luciferase activity compared to control.
