# Supplementary material for: TCF21 and the environmental sensor aryl-hydrocarbon receptor cooperate to activate a pro-inflammatory gene expression program in coronary artery smooth muscle cells
Source: PLoS Genet. 2017 May 8;13(5):e1006750. doi: 10.1371/journal.pgen.1006750 (PMC5439967; doi:10.1371/journal.pgen.1006750)
Supplement: S11 Fig — (PDF) [file pgen.1006750.s020.pdf]

Fig. S11

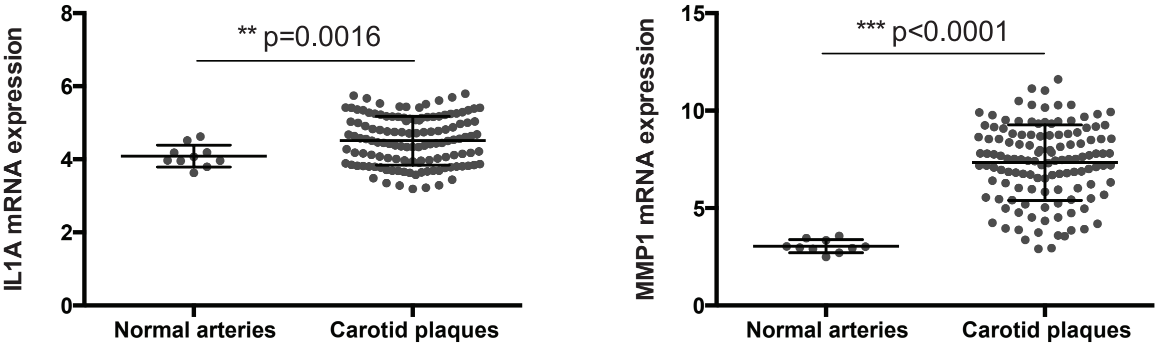

Figure S11. Increased expression of IL1A and MMP1 in human carotid artery plaques
BIKE biorepository data showing increased expression of *IL1A* and *MMP1* in human carotid artery plaques collected during endarterectomies compared to normal arteries (*IL1A* 3.04±0.10 vs. 7.33±0.17, $p<0.0001$; *MMP1* 4.09±0.10 vs. 4.51±0.06, $p=0.0016$). (** $p<0.005$, *** $p<0.0005$)
