# Supplementary material for: TCF21 and the environmental sensor aryl-hydrocarbon receptor cooperate to activate a pro-inflammatory gene expression program in coronary artery smooth muscle cells
Source: PLoS Genet. 2017 May 8;13(5):e1006750. doi: 10.1371/journal.pgen.1006750 (PMC5439967; doi:10.1371/journal.pgen.1006750)
Supplement: S12 Fig — (PDF) [file pgen.1006750.s021.pdf]

Fig. S12

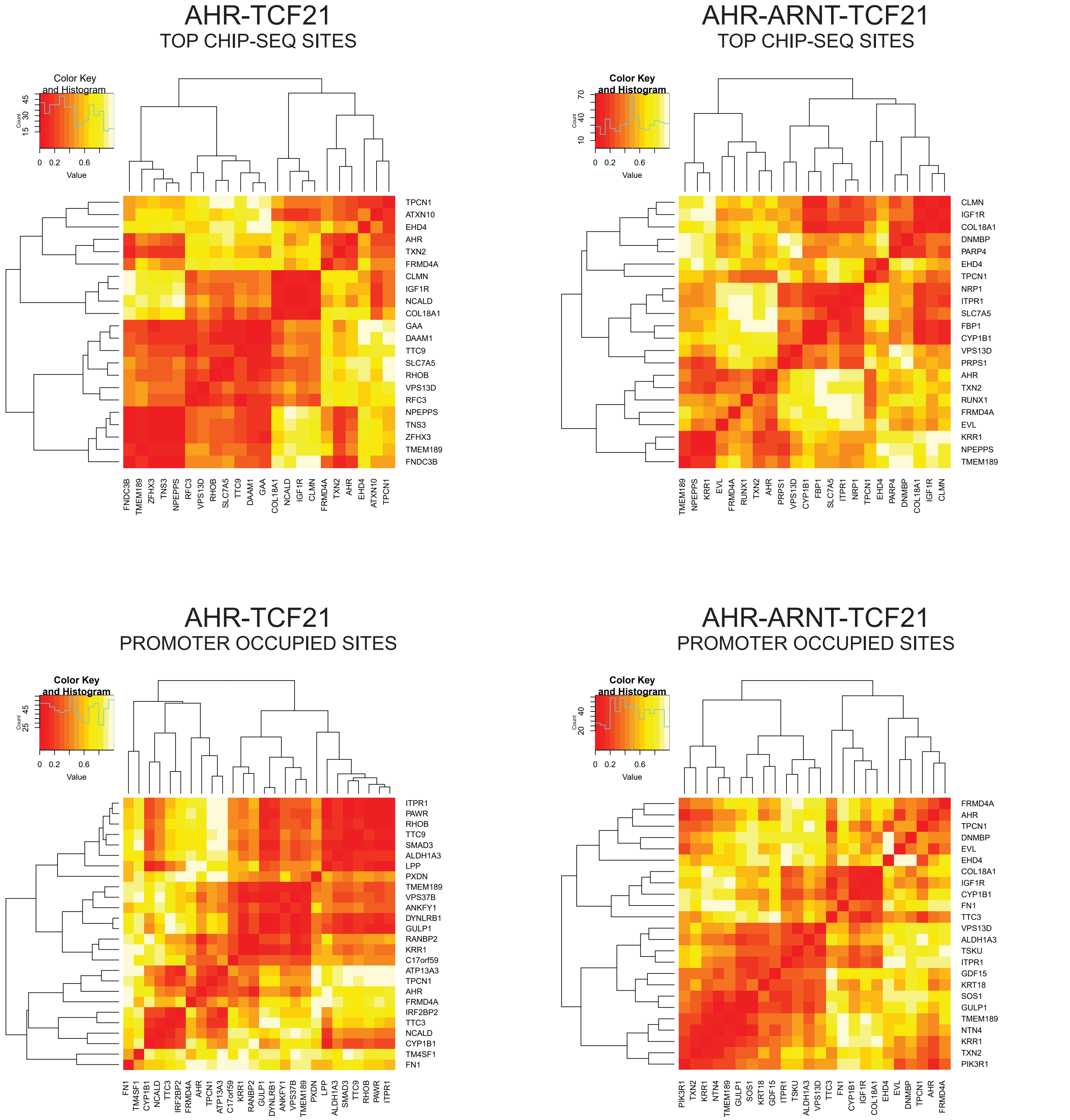

Figure S12.

AHR-TCF21 ChIP-Seq co-occupied genes display clustering patterns in mass-spectrometry and high resolution isoelectric focusing proteomic data from human carotid artery plaques. Proteomic datasets were constructed from 10+10 strictly phenotyped patients with asymptomatic and symptomatic carotid stenosis (males, statin users, age matched, plaques selected on CT and histology criteria). The lesions were analysed using mass-spec (MS) and high resolution isoelectric focusing with a yield of around 8-9000 recovered proteins. Dissimilarity index was created using the method that best discriminates all correlated pairs, given the formula: Dissimilarity = 1 - Abs(Correlation). Distance matrix was then created from the dissimilarity index. Clustering was done with heatmap.2 in gplots. Correlation matrix clustering based on (a) the top scored ChIPseq overlaps of AHR-TCF21 or (b) AHR-ARNT-TCF21 sites and (c) proximity of the co-occupied sites to the gene promoter regions for AHR-TCF21 co-occupied sites and (d) AHR-ARNT-TCF21. In (b) AHR clusters with RUNX1 transcription factor, while its target CYP1B1 co-clusters with extracellular matrix genes FN1, COL18A1 and with growth factor receptor IGF1R, emphasizing AHR and its downstream targets in regulation of extracellular matrix component of the human carotid artery plaque development.
