# Supplementary material for: TCF21 and the environmental sensor aryl-hydrocarbon receptor cooperate to activate a pro-inflammatory gene expression program in coronary artery smooth muscle cells
Source: PLoS Genet. 2017 May 8;13(5):e1006750. doi: 10.1371/journal.pgen.1006750 (PMC5439967; doi:10.1371/journal.pgen.1006750)
Supplement: S13 Fig — (PDF) [file pgen.1006750.s022.pdf]

Fig. S13

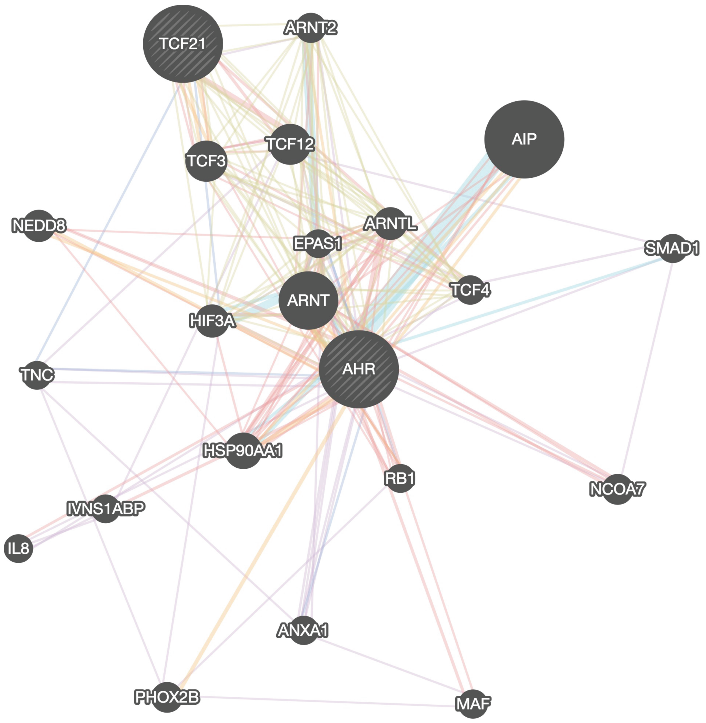

Figure S13. Network of interactions of TCF21 and AHR genes
Network was generated using Genemania tool and shows 231 total links based on protein-protein interactions, co-expression, predicted interactions, pathway anaysis, co-localization, genetic interactions and shared protein domains. TCF21 and AHR are connected in protein-protein interaction network through TCF4, TCF3, TCF12 and RB1.
